# Dimensionality and factorial invariance of religiosity among Christians and the religiously unaffiliated: A cross-cultural analysis based on the International Social Survey Programme

**DOI:** 10.1371/journal.pone.0216352

**Published:** 2019-05-15

**Authors:** Carlos Miguel Lemos, Ross Joseph Gore, Ivan Puga-Gonzalez, F. LeRon Shults

**Affiliations:** 1 Institute for Religion, Philosophy and History, University of Agder, Kristiansand, Norway; 2 Virginia Modeling, Analysis and Simulation Center, Old Dominion University, Norfolk, VA, United States of America; 3 Institute for Global Development and Planning, University of Agder, Kristiansand, Norway; 4 Center for Modeling Social Systems at NORCE, Kristiansand, Norway; Coventry University, UNITED KINGDOM

## Abstract

We present a study of the dimensionality and factorial invariance of religiosity for 26 countries with a Christian heritage, based on the 1998 and 2008 rounds of the International Social Survey Programme (ISSP) Religion survey, using both exploratory and multi-group confirmatory factor analyses. The results of the exploratory factor analysis showed that three factors, common to Christian and religiously unaffiliated respondents, could be extracted from our initially selected items and suggested the testing of four different three-factor models using multi-group confirmatory factor analysis. For the model with the best fit and measurement invariance properties, we labeled the three resulting factors as “Beliefs in afterlife and miracles”, “Belief and importance of God” and “Religious involvement.” The first factor is measured by four items related to the Supernatural Beliefs Scale (SBS-6); the second by three items related to belief in God and God’s perceived roles as a supernatural agent; and the third one by three items with the same structure found in previous cross-cultural analyses of religiosity using the European Values Survey (ESS) and also by belief in God. Unexpectedly, we found that one item, belief in God, cross-loaded on to the second and third factors. We discussed possible interpretations for this finding, together with the potential limitations of the ISSP Religion questionnaire for revealing the structure of religiosity. Our tests of measurement invariance across gender, age, educational degree and religious (un)affiliation led to acceptance of the hypotheses of metric- and scalar-invariance for these groupings (units of analysis). However, in the measurement invariance tests across the countries, the criteria for metric invariance were met for twenty-three countries only, and partial scalar invariance was accepted for fourteen countries only. The present work shows that the exploration of large multinational and cross-cultural datasets for studying the dimensionality and invariance of social constructs (in our case, religiosity) yields useful results for cross-cultural comparisons, but is also limited by the structure of these datasets and the way specific items are coded.

## Introduction

Religion plays an important role in the lives of many individuals today, as it has throughout history. The closely related concept of “religiosity” is just as important. However, “religiosity” is complex and difficult to define, because its study crosses multiple disciplines that use different viewpoints to approaching the concept [1]. It is no surprise, then, that scholars from a wide variety of disciplines, including cognitive science, psychology, anthropology, sociology, economics, and political science, have explored ways of identifying and measuring the factors of religiosity. Psychologists have been working for decades to identify the dimensions of individual-level religiosity and devise scales for their measurement. Hill and Hood [2] presented an extensive review of more than one hundred scales for measuring a wide range of domains related to religiosity, such as religious orientation, religious experiences, concepts of god, moral values, religious coping, etc. [2, 3]. Campbell and Coles recognized religiosity and religious affiliation as “independent dimensions” and pointed out the need to study differences of religious attitudes and beliefs between the religiously affiliated and unaffiliated [4].

This multiplicity of measurement instruments is beneficial to the scientific study of religion, but these instruments also have drawbacks. Perhaps the most notable of these is that many religiosity scales are based on the assumption that respondents are religious and contain items that make little sense for the nonreligious.

The availability of datasets from large-scale multinational surveys such as World Values Survey (WVS) [5], the European Values Study (EVS) [6], the International Social Survey Programme (ISSP) [7] or the European Social Survey (ESS) [8], opens new possibilities for the empirical study of religion. One drawback of these surveys is that they were not devised to test particular theories or aggregate measurement scales like the ones mentioned above. They typically include questions that are general and simple to interpret, which necessarily yields coarse descriptions of religiosity. On the other hand, these surveys’ large, multinational and cross-cultural samples, multiple time points and diversity of items permit comparative studies of religiosity across cultures and over time. Moreover, these datasets facilitate research on the relationship between religiosity and other social dimensions (moral values, social and political trust, attitudes towards minorities, etc.).

The statistical analysis of large cross-cultural datasets poses particular challenges. First, it is necessary to infer which meaningful dimensions (constructs, latent variables or factors) can be extracted from the data and which variables (items or indicators) measure each of them. This can be done by selecting variables according to theory, and using exploratory factor analysis (EFA) techniques to check that the factors and their indicators are meaningful [9–12]. The resulting factors can then be tested for measurement invariance, using multi-group confirmatory factor analysis (MGCFA) [13–16]. Comparisons between groups (and/or over time) are meaningful only if systematic measurement errors can be considered negligible.

Recently, some cross-cultural and longitudinal studies on measurement invariance using structural equation modeling (SEM) have been carried out in different areas (as shown in e.g. [17, 18]), some of them related to religion [19–22]. However, we are not aware of any previous work focused on the identification of universal factors of religiosity and analysis of their measurement invariance based on a large-scale multinational dataset, for both the religiously-affiliated and the unaffiliated. This latter aspect is particularly important for the study of secularization.

In this article, we attempt to contribute to this literature by describing a study of cross-cultural dimensionality (factor structure) and measurement (factorial) invariance for Christian-affiliated and religiously unaffiliated respondents from 26 countries with a Christian heritage, based on a set of selected items in the ISSP Religion Cumulation dataset from the 1998 and 2008 rounds. Our research questions are:

1. Which dimensions/factors of religiosity can be derived from the ISSP Religion Cumulation dataset, and how can these factors be related to dimensions of religiosity found in previous studies?
2. Are the derived factors invariant across gender, age, educational degree, religious group (Christian-affiliated or unaffiliated), and country?

We restricted our study to Christian (Roman Catholic, Protestant, Christian Orthodox and Other Christian Religions) and religiously unaffiliated respondents for a number of important reasons. First, with the exceptions of Israel and Japan, the countries included in the ISSP religion surveys are historically rooted in the Christian tradition. This was expected to introduce significant sample bias towards Christian religion. Second, several items in the ISSP Religion questionnaire are strongly associated with Christian religion and likely to have different meaning across religions (particularly for Hinduism and Buddhism). This potentially introduces construct biases [14, 23]. Finally, recent research on major religions has shown that in the next decades Christianity is expected to have the largest net loss from switching to the unaffiliated [24]. Thus, we were also interested in studying whether the differences between Christian-affiliated and the religiously unaffiliated are due to different latent means or factor scores of the same constructs, or (more profoundly) to different structural models.

The work was performed in three stages. First, we selected a set of items from the ISSP Religion Cumulation data based on on previous theoretical and empirical studies on the dimensions of religiosity [25–28], followed by the inspection of missing values, and by the application of variable transformations to allow for reliable computation of the correlation structures. In the second stage, we used EFA to confirm the expected number of factors and to identify which theoretically sound measurement models for these factors should be tested using MGCFA, using the 1998 data. Finally, we performed MGCFA analyses of measurement invariance for four three-factor models, based on the hierarchy of invariance levels introduced by Meredith [13] and described by many other authors (e.g. [14, 16, 23, 29, 30]), using the 2008 data. These tests were done separately for each of the following groupings: sex, educational degree, age, religious (un)affiliation group and country. We did these tests because previous studies showed that there are significant differences of religiosity across all these sociodemographic variables. We further explored the best-fitting of the four models by testing it for the invariance of latent means and factor variance-covariance structure across gender, age, educational degree and religious (un)affiliation group. The tests for measurement invariance confirmed that the best fitting model obtained in the EFA also had the best fit measures for the metric- and scalar-invariant models, particularly across the religious (un)affiliation groups and countries.

The remainder of this article is organized as follows. In the next section, we present a review of the empirical studies on the dimensionality and measurement of religiosity, that guided our selection of items from the ISSP and provided an initial clue on the number of dimensions expected in the EFA. In the Materials and methods section, we describe the procedures for preparation of the two data frames used for EFA and MGCFA, followed by the presentation of the methods used in the present work. The Results section contains a description of our findings on the “core” factors of religiosity derived from the ISSP Religion dataset, and on their invariance properties. In the Discussion section we compare our results with previous findings and discuss the theoretical contributions as well as the limitations of the present work. Finally, we present a summary of the main conclusions.

## Theoretical background

Research on the dimensions of religiosity can be traced to the turn of the twentieth century, when beliefs (core), religious works (morals), practices (rituals) and feelings (emotions) were already being distinguished as separate categories [31]. Empirical research in the past decades has shown that religion is a multidimensional construct [3, 25, 26, 28]. However, there is no general consensus on the number or nature of the dimensions of religiosity.

### Scales for measuring religiosity

The literature on the different dimensions of individual religiosity and the scales for their measurement is extremely vast. Here, we will present a summary review of the previous works that we found most useful for explaining our goals and methodology.

In 1965 Glock and Stark proposed five dimensions of religiosity: “belief”, “practice”, “experience”, “knowledge”, and “consequences” [25], which were later reduced to four by dropping the “consequences” dimension [27]. Other authors used EFA techniques to confirm the four-dimensional model of Glock and Stark, mostly based on samples of undergraduate students (e.g. [32, 33]). Jong and Halberstadt [21] reviewed subsequent studies on dimensions of religiosity inspired by the Glock and Stark model. It is noteworthy that none of these studies established the universality and cross-cultural validity of the model.

The Religious Orientation Scale (ROS) proposed by Allport and Ross [26] was designed to measure two dimensions of religious orientation: “intrinsic” (I) and “extrinsic” (E) religious orientations. Since its inception, many authors contributed to revise and improve the ROS (e.g. [9, 22, 34–39]). The most commonly used version is the “Age-Universal” ROS [9, 35]. Later, using EFA, the E-dimension was found to split into two factors, “social extrinsic” (Es) and “personal extrinsic” (Ep) [36, 37, 40]. Using confirmatory factor analysis (CFA) on a Polish sample of university students, Brewczynski and MacDonald showed that the ROS-based I-Es-Ep model is superior to the two-factor I-E model [41].

Batson [42] complemented the ROS by adding a third dimension called “Quest”, and Batson, Schoenrade and Ventis proposed a scale for its measurement [43]. The “Quest” scale is intended to measure readiness to face existential questions, perceptions of religious doubt and openness to change.

More recently, Saroglou [28] proposed a model he called “The Big Four Religious Dimensions” with the following four dimensions: “Believing” (cognitive), “Bonding” (emotional), “Behaving” (moral) and “Belonging” (social). This model builds on previously proposed descriptions of the dimensions of religiosity (see [28], Table I), particularly the simpler classification “beliefs”, “practice”/“behaving”, and “affiliation”/“identity” proposed by David Voas [44]. In Saroglou’s model, “Believing” refers to belief in some kind of transcendence (god(s), impersonal divinities, or transcendental forces or principles). “Bonding” captures the emotional effect of rituals, either public (worship, participation in religious ceremonies, etc.) or private (prayer and meditation). “Behaving” is related to moral behavior associated with religion, such as heightened altruism, sacrifice and humility relative to the wider social context, and taboo-conditioned behavior. Finally, “Belonging” refers to self-identification with a religious denomination or group. In collaboration with researchers from several countries, Saroglou developed the Four Basic Dimensions of Religiousness Scale (4-BDRS) for measuring the four factors. This scale was studied using samples of university students from Italy, the Netherlands and Mexico [45].

While the scales mentioned above are multidimensional, other authors have focused on a single dimension of religiosity. For example, the Supernatural Beliefs Scale (SBS) [21, 46, 47] was introduced “to measure the respondent’s tendency to believe in the existence or reality of supernatural entities, with minimal use of jargon from specific religions” (Jong and Halberstadt [21]). The original SBS consisted of ten items [46, 47] but was later reduced to six items for measuring respondents’ beliefs in God, angels and demons, soul, afterlife, existence of a spiritual realm and supernatural events (miracles). The SBS-6 was developed to be cross-culturally applicable, by structuring the items in the form of simple propositions that can be modified for different religious contexts (Muslim, Buddhist, Hindu, Sikh and Jainist populations) without introducing significant construct biases [21]. This scale was shown to be unidimensional using EFA, and its reliability and validity were confirmed for different cultural and religious contexts using samples from Brazil, Philippines, Russia and South Korea [21]. The development of the SBS-6 illustrates the need for using scales with few items of straightforward interpretation and wide cultural significance in multinational and cross-cultural studies.

### Limitations of religiosity scales

Despite their importance, the studies mentioned above have a number of significant limitations. First, many of them were based on samples of university students, often from just one or a few countries, which potentially introduces sample bias. Second, most scales were designed for Christian contexts and assume that the respondent is a religious person (the SBS-6 being an exception). In the “Age-Universal” ROS, for example, items IR.1—“I enjoy reading about my religion”, IR.4—“I try hard to live all my life according to my religious beliefs” and IR.5—“Although I am religious, I don’t let it affect my daily life” make little sense for nonreligious respondents. Likewise, in the 4-BDRS, items 1. “I feel attached to religion because it helps me to have a purpose in my life” and 10. “In religion, I enjoy belonging to a group/community” make little sense for those not attached to religion and unaffiliated to religious groups.

In addition, the scales’ items may fail to discriminate between religious and nonreligious individuals. For example, it is plausible to assume that both atheists and firm believers are likely to score low in item 10—“I am constantly questioning my religious beliefs” of the “Quest” scale, but for different reasons. Nonreligious persons may score high on item 6. “Religion has many artistic, expressions and symbols that I enjoy” in the 4-BDRS, without feeling a bond to religion.

One further limitation of the ROS and 4-BDRS is that their items related to attendance to regular services tap *purposes* (ROS) or *subjective evaluations* (4-BDRS) of the psychological effects of religious rituals, rather than *frequency* of participation (like in Glock and Stark’s scale for the “Religious practice experience” [25, 48]). Although subjective perceptions and judgments are essential to measuring religiosity, many scales lack items for quantitative expression of religious practices.

Finally, many studies using religiosity scales were based only on EFA, and few have addressed the scales’ universality and measurement invariance properties based on sufficiently representative samples.

### Measurement invariance studies of religiosity based on large surveys

The availability of datasets of large-scale multinational surveys [5–8] offers unique opportunities for cross-cultural and longitudinal studies of religiosity. The items on religion in these datasets are simple and straightforward to interpret and do not presuppose that the respondents are religious. Thus, dimensions found by analyzing these datasets will apply to both the religiously affiliated and unaffiliated. Moreover, because of their large, heterogeneous and cross cultural samples, these datasets are suitable for studying the dimensions of religiosity and their measurement (factorial) invariance across different countries and cultures.

Recently, Meuleman and Billiet [19] and Meuleman [49] used the ESS to investigate a potential factor of “religious involvement” measured by three items, one related to self-image as a religious person (“Regardless of whether you belong to a particular religion, how religious would you say you are?”), one to frequency of attendance to regular religious services (“Apart from special occasions such as weddings and funerals, about how often do you attend religious services currently?”), and another to frequency of praying (“Apart from when you are at religious services, how often, if at all, do you pray?”). Meuleman and Billet showed that this “religious involvement” factor met the criteria for partial metric invariance for the 25 countries, and partial scalar invariance for 21 out of 25 countries studied [19]. In particular, “religious involvement” in Turkey was found to be different from that in the other countries, due to the fact that the majority of the Turkish population is Muslim and attendance at religious services in Islam differs significantly between women and men. The latter study highlights how large data sets may help discovering dimensions of religiosity and identifying differences between countries and cultures.

### Summary

The above review can be summarized as follows:

- Individual religiosity is a multi-dimensional construct, but there is no general consensus on the number and meaning of these dimensions;
- Many authors have proposed scales for measuring one or more dimensions of individual religiosity. EFA has often been used to assess the dimensionality and internal validity of scales developed according to theory. In some studies, CFA has been used for confirmation of the models suggested by EFA and testing for measurement invariance;
- Most of the scales proposed for measuring individual religiosity were designed under the assumption that respondents are religious, and contain items with terms that require specific subjective interpretations. This limits their usefulness for large-scale, cross-cultural and multinational analyses;
- Items on religion in multi-national surveys [5–8] were not devised according to any particular theory, but are easily interpretable and meaningful for both the religiously-affiliated (in our case, Christian-affiliated) and unaffiliated. Thus, any dimensions found by analyzing these datasets are likely to have important and universal meaning.

With this background in mind, we used the ISSP Religion Cumulation dataset to find which dimensions could be extracted from it that may hold for both religious and nonreligious people. We also discussed the theoretical significance of our findings in relation to previous works and analyzed their universality and measurement invariance.

## Materials and methods

The ISSP Religion Cumulation dataset [50] contains the cumulated variables of the ISSP “Religion” surveys of 1991, 1998 and 2008 and comes in two separate files: a main file (ZA5070) with items and background variables that appear in at least two survey rounds, and an add-on file (ZA5071) with items that could not be cumulated for various reasons. The analysis in this article is based on the information in the main ZA5070 file, which includes 122 items for 102454 respondents from 28 countries. Details on the contents, structure and coding of the ZA5070 cumulation file can be found in [51]. The data processing was done using R [52]. S1 Table in the Supporting Information shows a list of the functions available in R packages used for performing the analysis reported herein [52–58].

### Data preparation

S2 Table in the Supporting Information shows the number of countries and respondents, as well as the % of respondents of each religious affiliation, for the three rounds in the ISSP Religion Cumulation dataset. The groups ‘Hinduism’, ‘Other Eastern Religions’, ‘Other Religions’ and ‘No (Christian) denomination’ given were eliminated because they were represented by residual proportions and also raised other problems such as possible construct biases in the case of ‘Hinduism’ or imprecise designation in the other cases. Respondents affiliated to Islam were removed from the analysis because they were mostly from Israel and were a minority in all countries represented in the dataset. It is also known that the relation between religious involvement and practice (praying and attendance to regular services services) is different between Muslims and Christians [19].

Jewish- and Buddhist-affiliated respondents were mainly from Israel and Japan, respectively, where each of these religions has strong historical roots. We excluded Jewish respondents from the analysis to keep the focus on just one major religion, and also because the 1998 data do not include information on attendance to regular religious services for Israel. Buddhist-affiliated respondents were also removed from the analysis, because some Buddhist religious groups were not represented in the ISSP rounds, and because some items related to beliefs in God, heaven and hell, and the relationship between God and meaning of life, could be affected by item and/or construct biases. Moreover, as a result of these decisions, Israel and Japan were dropped from the analysis, because Christian respondents were a small minority in both countries.

**Fig 1:**
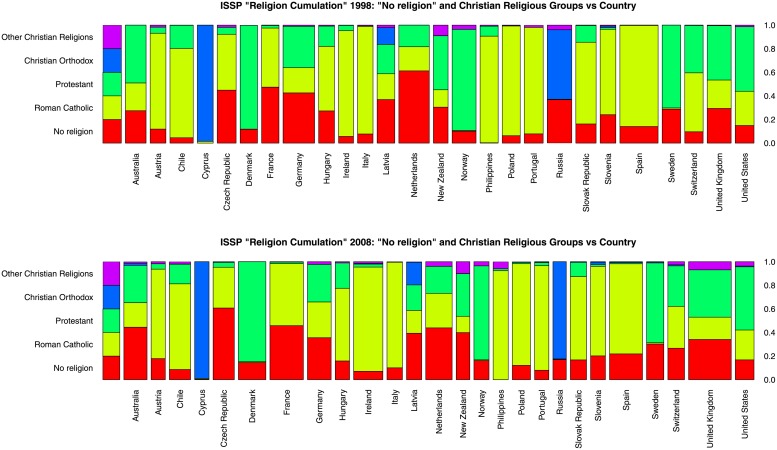
Christian and religious unaffiliated by country, years 1998 and 2008. Proportions of Christian-affiliated and religiously unaffiliated respondents by country for years 1998 (top) and 2008 (bottom), based on [50].

Table 1 shows information on the countries, the number and the percentage of Christian-affiliated and religiously unaffiliated respondents in the three rounds of the ISSP Religion Cumulation dataset (after removing the religious groups and countries mentioned above). Fig 1 shows spine plots of the distributions of Christian-affiliated and ‘No religion’ respondents for the 26 countries considered in our analyses.

**Table 1:** Number of countries, and number and % of Christian-affiliated and religiously unaffiliated respondents in the 1991, 1998 and 2008 rounds of the ISSP Religion questionnaire (Israel and Japan excluded).

	1991	1998	2008
Number of countries in dataset	15	26	26
Number of respondents in dataset	22944	33129	36669
No Religion (%)	25.65	22.83	25.09
Roman Catholic (%)	41.13	45.04	44.23
Protestant (%)	28.78	24.76	23.02
Christian Orthodox (%)	4.01	6.26	5.70
Other Christian Religions (%)	0.26	1.10	1.96

**NOTE**: The religious affiliations correspond to the categories of the RELIGGRP background variable in the ISSP Religion Cumulation dataset [50].

Since EFA and CFA must be run with independent data [10], we first split the original file into two data sets, one with the data from year 1998 and another from year 2008. This automatically ensured independence between the two data sets. However, in doing this we had to assume that the configural model of the “core” factors of religiosity was longitudinally invariant in the 10-year period from 1998 to 2008. Other alternatives, such as random sampling of the data, would introduce some degree of dependence of the data sets used for EFA and MGCFA. Analyzing the three waves separately in an attempt to determine longitudinal variations would reduce the sample sizes, particularly for minority groups, and increase the problem of missing values due to lack of item information across rounds. In this work we did not attempt to study longitudinal invariance of factors via CFA, which would require a slightly different approach than for cross-cultural analyses (see e.g. [59]). Data from the 1991 round were not used because that round included fewer countries than the 1998 and 2008 rounds.

In addition, we eliminated records of respondents with one or more sociodemographic values missing, and then records with more than five values of selected items missing. The resulting data frames included 97.5% (32297 records) and 96.8% (35513 records) of the 1998 and 2008 data frames, respectively.

#### Variables’ selection

Table 2 shows the selected items and sociodemographic (background) variables used in the present work and included in the 1998 and 2008 data frames. Age was categorized using the Harmonized Standard 2 of the UK Office for National Statistics [60], so that it could be used as a grouping variable. Next, we will present our rationale for selecting the items shown in this table based on the literature review above.

**Table 2:** Selected items and sociodemographic variables. Selected items and sociodemographic (background) variables for the Christian-affiliated and religiously unaffiliated respondents (Table 1) with complete sociodemographic information and at most five missing items, in the 1998 and 2008 rounds in the ISSP Religion Cumulation dataset. The sociodemographic variables are listed in the “Item” column below the thick line after ATTEND.

Item	Question label	Type	Levels*	% missing (1998)	% missing (2008)
V28	Please indicate which statement comes closest to expressing what you believe about God.	nominal	6 (3)	0.88	0.78
V30	Do you believe in life after death?	ordinal	4	12.31	8.41
V31	Do you believe in heaven?	ordinal	4	13.08	8.42
V32	Do you believe in hell?	ordinal	4	13.85	9.22
V33	Do you believe in religious miracles?	ordinal	4	12.66	7.00
V35	Agree/Disagree: There is a God who concerns Himself with every human being personally?	ordinal	5	7.57	6.15
V37	Agree/Disagree: To me, life is meaningful only because God exists.	ordinal	5	4.70	3.66
V49	About how often do you pray?	ordinal	11 (5)	1.33	1.85
V50	How often do you take part in the activities of organizations of a church or place of worship other than attending services?	ordinal	11 (5)	0.75	0.97
V51	Would you describe yourself as religious?	ordinal	7 (5)	2.11	1.93
ATTEND	How often do you attend religious services?	ordinal	6 (4)	5.51	3.98
AGE	Age group of respondent	ordinal	5**	–	–
SEX	Sex of respondent	nominal	2	–	–
DEGREE	Highest education level/degree of respondent	ordinal	6	–	–
RELIGGRP	Religious main group	nominal	12	–	–
COUNTRY.NAME	Country name	nominal	26	–	–

* The values shown within parentheses are the variables’ number of levels after the transformations described below;

** The numeric variable AGE in the ZA5070_v1-0-0.RData data file was converted into an ordinal variable with the following categories (age groups): 0-24, 25-44, 45-64, 65-74, 75+ These correspond to the Harmonized Standard 2 of the UK Office for National Statistics.

Item V28 “Please indicate which statement comes closest to expressing what you believe about God” is intended to measure belief in God, which is a key factor of an individual’s religiosity in almost all theoretical models (e.g. [25, 26, 28, 44, 61, 62]). Item V29 in the ISSP Religion Cumulation dataset, “Which best describes your beliefs about God?”, is also related to belief in God. However, it was not selected because its levels (“I don’t believe in God now and I never have”, “I don’t believe in God now, but I used to”, “I believe in God now, but I didn’t used to”, and “I believe in God now and I always have”) are related to *changes* of belief and do not express the *level* of belief in a clearly ordinal scale.

Items V35 “Agree/Disagree: To me, life is meaningful only because God exists” and V37 “Agree/Disagree: There is a God who concerns Himself with every human being personally?” can be related to items one and two of the 4-BDRS for measuring the “Belief” dimension, although there are important differences between the ISSP and 4-BDRS items. In the 4-BDRS, the association is between religion and life’s purpose, and between “Transcendence” and “meaning to human existence”, whereas in the ISSP the associations are between God, protection and life’s meaning. Despite these differences, we nevertheless expected that the two items in the ISSP would form a factor together with the one mentioned above (expression of belief in God).

The four items V30–V33 “Do you believe in life after death?”, “Do you believe in heaven?”, “Do you believe in hell?” and “Do you believe in religious miracles?” measure general beliefs in supernatural phenomena rather than God (a supernatural agent): survival of death, supernatural reward, supernatural punishment and supernatural events/intervention. In addition, these beliefs are central to the doctrines of the Christian faith [63]. Based on the theoretical formulations and empirical evidence behind the SBS-6 mentioned above [46], we hypothesized that these items would form a factor.

Items V50 “How often do you take part in the activities of organizations of a church or place of worship other than attending services?”, V51 “Would you describe yourself as religious?” (which Campbell and Coles call the self-rated religiosity, [4] Table 1) and ATTEND “How often do you attend religious services?” measure the current religious involvement of respondents. They are related to the “Religious practice” dimension in the Glock and Stark model (see e.g. [48]), and partly to item 5. in the 4-BDRS. Item V49 “About how often do you pray?” is also related to religious practice. However, prayer can be collective or individual, and this distinction is not clear in the ISSP questionnaire. In addition, prayer can serve both individual and social psychological functions [61, 64], so we were not sure in which factor this item might load. Since previous cross-cultural analyses based on the ESS considered a “religious involvement” factor consisting of three items with similar meaning (self-image as a religious person, and frequencies of attendance and praying) [19, 49], we were interested in confirming whether a factor with similar structure and meaning could also be found in the ISSP dataset.

The ISSP Religion dataset includes many other items that are important for the scientific study of religion, such as attitudes towards sexual behavior and abortion, gender role in family life, moral attitudes in civil life, confidence in churches and other institutions, frequency of churchgoing by the respondents and their parents during the formers’ formative period, feelings about the Bible, paranormal beliefs, picture of God, social trust and world views, trust in science and religious conflict. However, these items are not directly related to the *core dimensions* of religiosity we identified in our comparative review of the literature, or do not refer to the respondent’s present condition. Moreover, many of them are likely to be strongly influenced by many other social, political and cultural factors that are not always explicitly (or only) religious. For these reasons, none of these items was considered in our analysis.

After presenting the rationale behind our selection of items, it is natural to ask: how many dimensions were expected to be found in the EFA? Based on the previous studies mentioned above, we expected to find either two or three factors. In the former case, the factors would be related to beliefs and current religious involvement, while in the latter case the beliefs factor would split into two factors related to God and afterlife, respectively. In either case, we were unsure about whether or not these dimensions were common to the Christian-affiliated and the religiously unaffiliated.

#### Missing values

Table 2 shows that the percentage of missing values for datasets used in the EFA and MGCFA ranged from 0.75% (for item V50) to 13.85% (for item V32). S1 Fig in the Supporting Information shows the missing data pattern for the 1998 data. This figure clearly shows that the missing values pattern is not Missing Completely at Random (MCAR) [65], so we did not perform Little’s test [66]. In the present work, we used pairwise-complete observations to compute the polychoric correlation matrices in EFA, and the default listwise deletion method in lavaan for the MGCFA, since the Full Information Maximum Likelihood (FIML) method implemented in lavaan cannot be used with ordinal data.

#### Variables’ transformations

Items were reverse-coded so that the top levels would correspond to the highest degrees of belief in God and afterlife, miracles, self-image as a religious person and frequency of religious practices (praying and attending regular church services). The numeric sociodemographic variable AGE was categorized and converted to an ordered factor, with the categories (age groups) shown in Table 2. The respondents’ highest education level (DEGREE) was also declared an ordered factor. We also merged the items’ levels to avoid categories with zero or very few counts or to obtain transformed items with clear ordinality, as described below.

The levels of item V28 “Please indicate which statement comes closest to expressing what you believe about God” are: “I don’t believe in God”, “Don’t know whether there is a God, don’t believe there is a way to find out”, “Don’t believe in a personal God, but I do believe in a Higher Power”, “I find myself believing in God some of the time, but not at others”, “While I have doubts, feel that I do believe in God” and “I know God really exists and have no doubts about it.” These levels do not express the level of belief in a clearly ordinal way, because the third level mixes the level of belief with the respondent’s view about God’s nature, and the distinction between levels four and five is not very clear. We therefore merged levels 2-5 into a single level “I have doubts about God.”

The frequency of the respondent’s attendance to regular religious services (ATTEND) is coded with the following levels: “Never”, “Less frequently,’ “Once a year”, “Several times a year”, “Once a month”, “2 or 3 times a month”, “Once a week” and “Several times a week.” However, the levels of this variable were defined differently in the 1998 and 2008 questionnaires [67, 68], and some levels contained very few counts. We therefore merged levels 2-4 into “Yearly”, 5 and 6 into “Monthly” and 7-9 into “Weekly.” We also merged the levels of items related to the respondent’s and his/her parents attendance to regular religious services during the respondent’s formative years into the same levels as for ATTEND.

The items related to the respondent’s frequency of praying (V49) and attendance to church activities other than regular services (V50) are coded with the following levels: “Never”, “Less than once a year”, “About once or twice a year”, “Several times a year”, “About once a month”, “2-3 times a month”, “Nearly every week”, “Every week”, “Several times a week”, “Once a day” and “Several times a day.” We merged the levels of these items as follows: 2-4 into “Yearly”, 5 and 6 into “Monthly”, 7-9 into “Weekly” and 10 and 11 into “Daily.”

Finally, we also transformed the item V51 “Would you describe yourself as religious?” with levels “Extremely non-religious”, “Very non-religious”, “Somewhat non-religious”, “Neither religious nor non-religious”, “Somewhat religious”, “Very religious”, “Extremely religious”, by merging levels 1 and 2 into “Highly non-religious” and 6 and 7 into “Highly religious.”

These transformations allowed more stable computations of the polychoric correlation matrices, which improved the quality of the EFA solutions and avoided convergence problems in the MGCFA tests.

### Exploratory factor analysis method

The EFA was used to confirm the expected number of factors and their indicators, and check whether or not all the latter had sufficient communality and loadings. However, the factor extraction methods are based on the assumption that the correlation structure comes from a homogeneous population, i.e. is not biased by group mean differences. This is generally not the case in large multinational datasets with largely heterogeneous distributions of sociodemographic variables. This problem can be overcome using multi-level factor analysis (MFA), in which three covariance (or correlation) matrices are used: the total covariance matrix **Σ**_*T*_, the between-group covariance matrix **Σ**_*bg*_, and the within-group covariance matrix **Σ**_*wg*_ [11, 12, 69, 70]. This method allows the determination of the proportions of the total variance due to group membership and to variation within each group, and whether or not the constructs’ meaning changes between the individual and aggregate (group) levels. Since our interest was the identification of factors at the individual level, we used this decomposition to obtain a pooled within-group correlation matrix **R**_*wg*_. This matrix was computed by weighting the polychoric within-group correlation matrices for each group, with weights proportional to the group size. This procedure removed the effect of group means shifts from the correlation structures and allowed the computation of factor solutions for the individual level only, based on **R**_*wg*_.

We started the EFA by identifying the sociodemographic variables that led to the sharpest group means differences of the selected items. For this, we used Chernoff faces plots [71] and the intra-class correlation coefficient ICC(1) (which describes the proportion of variance associated with the grouping variable [70, 72]) for qualitative and quantitative evaluations, respectively. We found that the sharpest differences between mean structures is due to the religious group, particularly the differences between Christians and the religiously unaffiliated. Based on this conclusion, we computed factor solutions for the corresponding pooled within-group correlation matrix **R**_*wg*_ = **R**_*w*.*RELIGGRP*_.

The factor solutions based on the **R**_*w*.*RELIGGRP*_ matrix were computed using the minimum residual method for factor extraction [56, 73] and squared multiple correlations (SMC) as communality estimates [73]. Since we expected the factors to be significantly correlated, we used the “oblimin” oblique rotation method [74]. Although theory strongly suggested that the selected variables would yield a three-factor model, we nevertheless needed to confirm the number of factors. This could be done using the scree test [75], the Very Simple Structure (VSS) [76] and Velicer’s Minimum Average Partial (MAP) criterion [77], and parallel analysis [78]. We used parallel analysis because it consistently yields correct estimates of the number of factors in many cases [79].

We checked the number of factors and confirmed the factors’ meaning in relation to the theoretical considerations behind the items’ selection. We also examined the fit measures and checked items for insufficient communality (<0.5) and loadings (maximum absolute value <0.32), cross-loadings (items with loadings with absolute value >0.32 in more than one factor), loadings with absolute value greater or equal to 1.0, and Heywood and ultra-Heywood cases (communality equal to 1.0 or greater than 1.0, respectively) [10].

### Multi-group confirmatory factor analysis method

We performed multi-group factorial invariance analysis of the model selected after the EFA part for the following groupings (units of analysis): sex, educational degree, age, religious (un)affiliation and country. MGCFA was based on the linear common factor model. Measurement invariance was studied by testing for configural, metric, scalar and strict invariance via a sequence of nested models with increasingly strong constraints [13, 14, 29, 80–82]. Since our model contains ordinal indicators, the identification conditions and the invariance constraints are different from those for models with continuous indicators. S1 Appendix contains a description of the identification and invariance constrains used in the present work, together with a summary of the relevant theoretical background [14–16, 29, 81, 83, 84]. The methods for parameter estimation, testing of the invariance hypotheses, and evaluating Goodness Of Fit (GOF) are described below.

#### Estimation method

Since our model involves ordinal items, we used the diagonally-weighted least squares (DWLS) estimator with mean and variance adjustment of the test statistic (also known as WLSMV [82, 85, 86]). We also used the Θ–parameterization, to allow testing for the invariance of the residual variances [29, 87].

#### Measurement and structural invariance tests

Following the general approach described in [13, 29, 82, 87], we performed tests of configural, metric (weak), scalar (strong) and strict invariance, in this order. In the cases where metric invariance was obtained, we tested for invariance of the factor variance-covariance structure across groups; in the cases where scalar invariance was obtained, we also tested for equality of the latent means across groups [15].

#### Fit evaluation

The methods for fit evaluation and the criteria for rejection of invariance hypotheses in measurement invariance studies have been the object of intense research [88–92]. The evaluation of model fit is based in the *χ*^2^ test statistic for the minimum of the fit function used to estimate the model parameters, which depends on the discrepancy between the observed and model-implied variance-covariance matrices. However, inferences based on the *χ*^2^ value often lead to artificial over-rejection for large sample sizes [88, 89, 93–96]. Likewise, likelihood-ratio tests (corrected *χ*^2^ difference tests) for comparing nested models are strongly affected by sample size and model complexity [91, 94]. Rutkowski and Svetina [95] report a study with varying number or groups in which they found that the *χ*^2^ differences increase with the number of groups and led to consistent rejection of metric and scalar invariance for fully invariant models ([95], page 45).

Therefore, numerous authors have recommended the combined use of several different indices for assessing model fit, and criteria for rejecting invariance hypotheses based on the degradation of these indices between consecutive nested models with increasing invariance constraints [10, 15, 88, 90, 97]. However, the criteria on the difference of fit between nested models have mostly been based on simulations with simple models (small number of factors and groups) with continuous indicators and maximum likelihood (ML) or robust ML estimation. Recently, Sass, Schmidt and Marsh found that application of these criteria to models with ordered items and WLSMV estimation may yield inflated Type I error rates [92]. In the present work, we adopted conservative criteria for model fit and rejection of invariance hypotheses.

Following the mainstream literature on measurement invariance, we based our assessment of model fit on the comparative fit index (CFI), the root mean square error of the approximation (RMSEA) and the standardized root mean square residual (SRMR), with cutoff values for acceptable fit 0.95, 0.06 and 0.08 respectively [88]. The description of these fit measures can be found in e.g. Schermelleh-Engel et al. [93] and Schumacker and Lomax [97]. Recently, the weighted root mean square residual (WRMR) index was proposed for assessing the fit of models with ordinal items. However, the usefulness of this index for testing measurement invariance has not been established [92, 98].

Several criteria have been proposed to test invariance hypotheses, based on how one or more fit indices change between successive nested models with increasing restrictions. The most commonly used criterion is to reject that two successive models are statistically equivalent is ΔCFI ≤ −0.01 [88]. We adopted the more stringent rejection criteria proposed by Chen [90] and by Meade et al. [91] for models with large samples: ΔCFI ≤ −0.002 (Meade et al. [91]) and ΔRMSEA ≥ 0.015 (Chen [90]) for both metric and scalar invariance tests; and ΔSRMR ≥ 0.030 and ΔSRMR ≥ 0.010 for metric and scalar/strict invariance tests, respectively (Chen [90]). S3 Table in the Supporting Information shows a summary of the GOF measures and cutoff criteria used in the present work.

In cases where the invariance hypotheses were rejected, we tried to improve the models’ fit by excluding the groups with largest *χ*^2^ contribution [99] and/or freeing parameters to attain partial invariance [100], depending on each particular situation.

## Results

### Exploratory factor analysis

To perform an EFA, we first needed to remove the effects of the differences between group means. In our case, the country was the obvious group variable (or unit of analysis). However, we expected that the variance associated with other sociodemographic variables such as the religious group could be just as important, if not more. Thus, before carrying on the EFA we analyzed the differences of mean structures for each sociodemographic variable, using qualitative and quantitative methods.

The qualitative analysis was done using Chernoff faces plots [71], in which the numerically-coded group means of the selected items were mapped into face features as shown in S4 Table in the Supporting Information. We are aware that means of numerically-coded ordinal variables cannot be used for quantitative inference. However, since this analysis was qualitative, we considered the procedure acceptable for our purpose. S2 and S3 Figs in the Supporting Information show the faces plots for the composite factor religious (un)affiliation/educational degree and for the 26 countries, respectively, for the 1998 data.

Clearly, group mean differences due to (un)affiliation are much more pronounced than those due to the educational level. The most salient difference is between the religiously unaffiliated and the Christian groups. Among the latter, the ‘Roman Catholic’ and ‘Other Christian Religions’ look similar in terms of their high mean levels of religious beliefs (hair, eyes and nose), rituals’ frequency (mouth) and self-image as a religious person (hears). The faces plots for religious (un)affiliation/gender and religious (un)affiliation/age lead to conclusions similar to those drawn from S2 Fig.

S3 Fig shows that countries are very heterogeneous with respect to their “overall religiosity.” Nevertheless, the countries’ religiosity can be partly explained by their respective shares of each religious group (Fig 1). For example, countries with a large proportion of ‘No religion’ or ‘Protestant’ respondents fill the top rows, while the highly religious countries have heterogeneous characteristics but are very different from the highly secular ones. In addition, the faces representing the Russia and Ireland in S3 Fig are remarkably similar to the ‘No religion’ and ‘Roman Catholic’ faces in S2 Fig, reflecting their very strong secularism and Catholic tradition, respectively.

**Fig 2:**
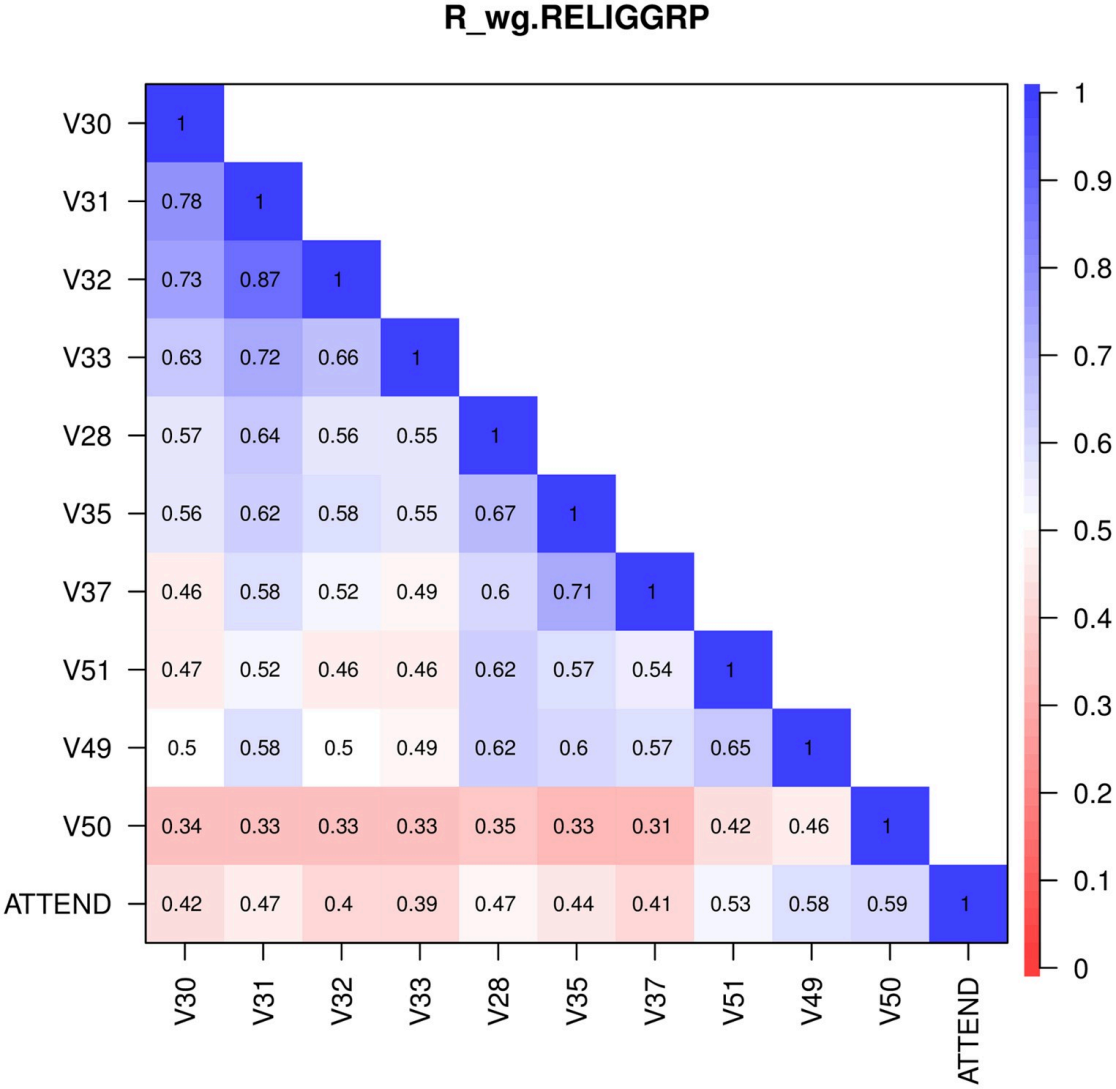
Pooled within-group correlation matrix. Pooled within-group correlation matrix **R**_*w*.*RELIGGRP*_, computed by weighting the within-group polychoric correlation matrices for the religious group (RELIGGRP), with weights proportional to group size, based on the 1998 ISSP Religion data [50].

Following the qualitative analysis using faces plots, we computed the intraclass correlation coefficient ICC(1) for the selected items and for each sociodemographic (grouping) variable, as shown in Table 3. For all items, the proportion of between-group variance for gender, age group and highest educational degree is very low. These groupings potentially introduce large pooling effects, so that the resulting tests (in the MGCFA stage) would have low power for rejecting hypotheses concerning measurement invariance. For most items, the proportion of between-group variance is highest for the religious (un)affiliation group, followed by the country, despite the former having only five groups and the latter 26. Therefore, we decided to perform an EFA based on the weighted pooled-within polychoric correlation matrix for the religious groups, **R**_*w*.*RELIGGRP*_. Fig 2 shows this correlation matrix.

**Table 3:** Intraclass correlation coefficient ICC(1). Intraclass correlation coefficient ICC(1) (proportion of the total variance due to group membership) for the selected items, for each of the following grouping variables: gender (SEX), age group (AGE), educational degree (DEGREE) and country (COUNTRY.NAME), based on the 1998 data [50].

	SEX	AGE	DEGREE	RELIGGRP	COUNTRY.NAME
V28	0.030	0.022	0.040	0.307	0.196
V30	0.039	0.004	0.005	0.143	0.121
V31	0.034	0.006	0.034	0.236	0.213
V32	0.015	0.003	0.024	0.186	0.197
V33	0.029	0.003	0.027	0.209	0.167
V35	0.034	0.013	0.031	0.272	0.185
V37	0.019	0.052	0.060	0.235	0.186
V49	0.078	0.044	0.039	0.313	0.187
V50	0.010	0.008	0.005	0.136	0.108
V51	0.039	0.037	0.025	0.360	0.127
ATTEND	0.023	0.029	0.023	0.296	0.201

We first ran an EFA based on **R**_*w*.*RELIGGRP*_ for the items in Table 2, as described in the Materials and methods section. The estimated number of factors was three, and this led to the best fitting solution. Inspection of the factor solution revealed that item V49 (“About how often do you pray?”) was cross-loading and item V50 (“How often do you take part in the activities of organizations of a church or place of worship other than attending services?”) had a borderline insufficient communality (*h*^2^ = 0.49). The correlation structure illustrated in Fig 2 also shows that the correlations between V50 and items V28, V30-V33, V35 and V37 are the weakest.

Owing to these problems, we tried removing item V50 (whose theoretical importance is lower than the frequency of church attendance or praying) and recomputing the resulting factor solution. For this second model, the estimated number of factors was again three. Inspection of the factor solution showed that item V49 was no longer cross-loading, but item V28 (“Please indicate which statement comes closest to expressing what you believe about God”) was cross-loading and the communality of item ATTEND (“How often do you attend religious services?”) was borderline insufficient in the ten-item model (*h*^2^ = 0.48). Since both V28 and ATTEND were of primary theoretical importance, we decided not to remove more items and proceed with further analysis of the two candidate models.

**Fig 3:**
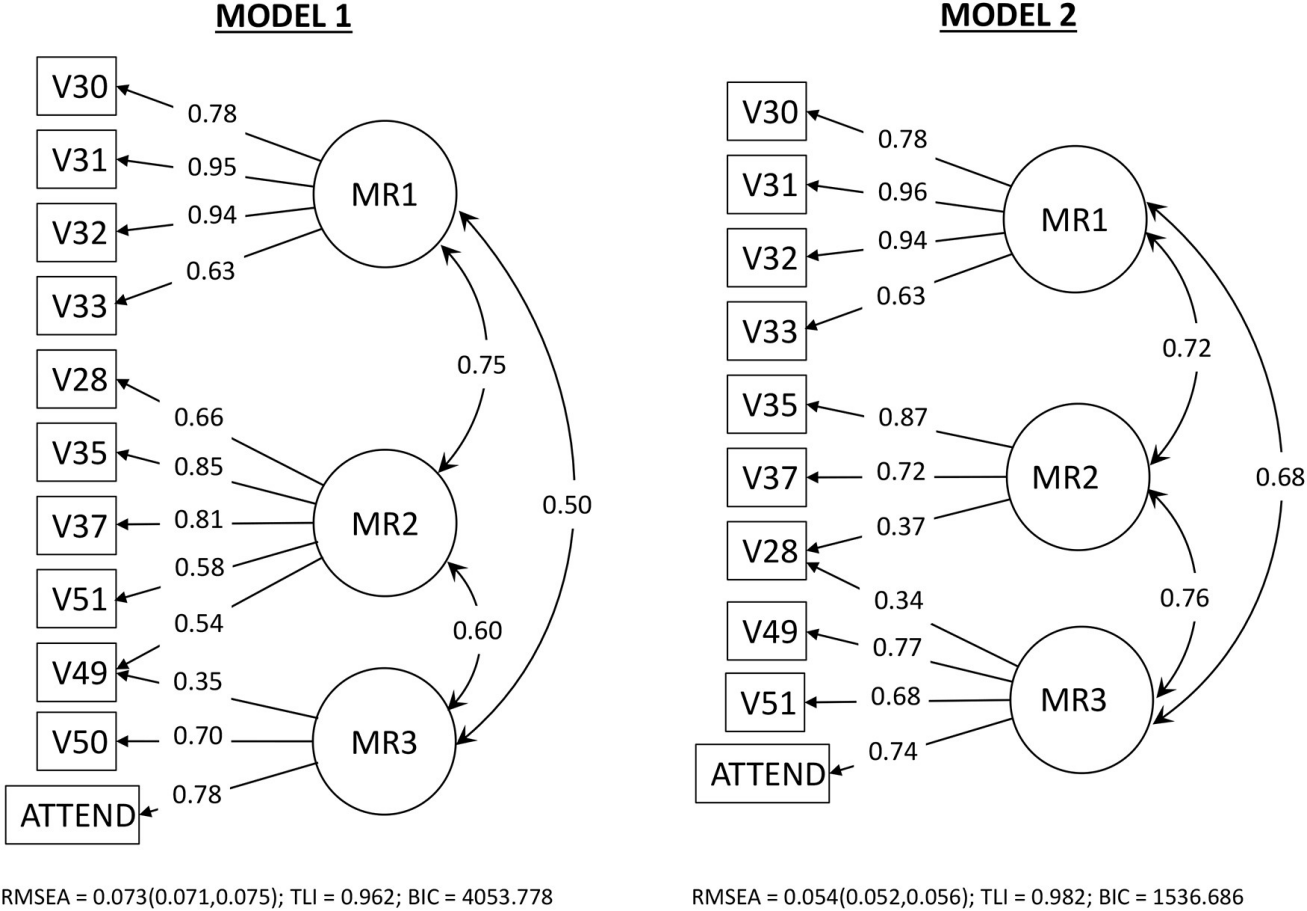
Factor solution diagrams. Solution diagrams for the two three-factor models based on the correlation matrix **R**_*w*.*RELIGGRP*_ computed using 10 and 11 items as described in the text and based on the 1998 ISSP Religion data. In this figure RMSEA is the mean square error of approximation, TLI is the Tucker-Lewis index and BIC is the Bayesian information criterion [56].

Fig 3 shows the solution diagrams for the first (eleven-item) and second (ten-item) factor models. In the first model, the factors MR1 and MR3 are interpretable as “Beliefs in afterlife and miracles” and “Religious practices”, respectively. However, the interpretation of the factor MR2 is not clear, because this factor’s items are very heterogeneous (belief in God, God’s roles as a supernatural agent, self-image as a religious person and one form of religious practice). Since the ISSP does not differentiate between private and public prayer (related to “intrinsic” beliefs and perceptions and to “extrinsic” rituals’ expressions, respectively), the cross-loading of item V49 could not be ruled out a priori as unlikely.

The second (ten-item) model is sounder from a theoretical viewpoint because the factors are easier to interpret. Factor MR1 is the same as in the eleven-item model. Factor MR2 is interpretable as “Belief and importance of God.” Except for the cross-loading item V28 (related to the level of belief in God), the three items in the factor MR3 bear close relationship with the corresponding items of the “Religious involvement” factor found in the ESS [19]. Apart from being theoretically sounder, the second model also has better fit measures (Fig 3). In particular, it is substantially more parsimonious than the first model, as is evident from it’s much lower BIC value.

In summary, the results of EFA suggest that Model 2 is superior to Model 1. We nevertheless tested both models for measurement invariance using MGCFA to confirm this conclusion. We also tested two congeneric variants of Model 2 which we called Models 3 and 4. In Model 3, we eliminated the item V28 from the measurement of the “Religious involvement” factor. In Model 4, we eliminated item V28 from the measurement of the “Belief and importance of God” factor and relabeled the resulting two-item factor simply as “Importance of God”. Although model 4 is not very plausible, we analyzed it to understand how different ways of removing the cross-loading of item V28 would affect the results of the invariance tests.

### Confirmatory factor analysis

We first ran measurement invariance tests for the four models described in the previous section, across the grouping variables SEX (gender), AGE (age group), DEGREE (highest educational degree), RELIGGRP (religious group), and COUNTRY.NAME (country). We had to remove Denmark and Russia to perform the measurement invariance tests for the countries owing to zero counts in the top level of item V49 (frequency of prayer). Based on the results in Table 3, we expected that any lack of measurement invariance, particularly at the scalar level, would be detected when testing across the religious groups or the countries (because of the large between-group variance for these two units of analysis).

S5–S8 Tables show the results of the measurement tests for Models 1–4, respectively. S5 Table shows that Model 1 led to invalid solutions for the metric-invariant (constrained thresholds and loadings) model for the religious group, and for both the metric- and scalar-invariant (constrained thresholds, loadings and intercepts) models for the countries. This confirmed that Model 1 is clearly inferior to Model 2, as was expected from its worse fit measures obtained in the EFA solutions (Fig 3). Thus, we made no further attempts to improve Model 1 and concentrated on the analysis of Model 2 and its variants.

**Fig 4:**
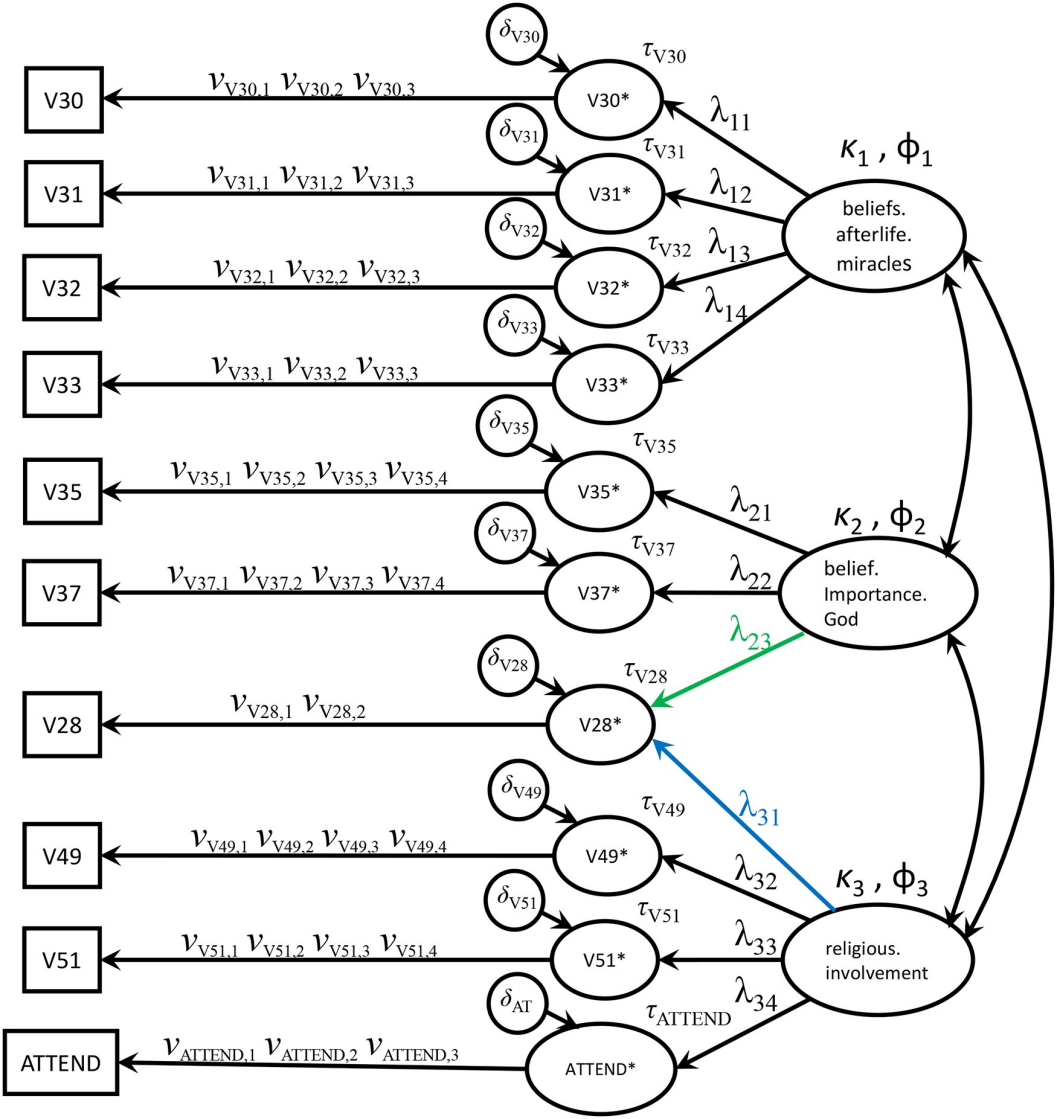
Path diagrams. Path diagrams for Models 2, 3 and 4 referred in the text. Model 2 includes both the green and blue loadings and corresponds to the ten-item model suggested by the EFA. Model 3 only includes the green loading. Model 4 only includes the blue loading.

Fig 4 shows the path diagrams for Models 2, 3 and 4. S6 Table shows that Model 2, which corresponds to the ten-item factor solution obtained with EFA is the best fitting of the four models. It is the only model that according to our criteria yields up to scalar invariance across the religious (un)affiliation groups. For the tests across the countries, which are the most stringent, we rejected the hypothesis of metric invariance based on the excessive value of RMSEA (0.062, with the 90% confidence interval above 0.06). Models 3 and 4 also led to rejection of metric invariance across the countries but with worse RMSEA than Model 2. This provided some evidence that the model suggested by the EFA (Model 2) is better than the two congeneric models obtained by removing one of the regression paths for the cross-loading item V28. Thus, we proceeded by trying to improve the fit of Model 2 for the countries.

Table 4 repeats the information in S6 Table and also shows the results of our attempts to obtain metric and scalar invariance across the countries. To improve the fit of the metric-invariant model we removed The Netherlands (the country with the highest *χ*^2^ contribution). This led to a model with constrained thresholds and loadings that met our criteria for accepting the hypothesis of metric invariance. The scalar-invariant model across the remaining 23 countries had poor fit and was considerably more difficult to improve.

**Table 4:** Model 2: GOF indices for the measurement invariance tests. Estimates of the GOF indices for the measurement invariance tests for gender, age group, highest educational degree, religious (un)affiliation and initial set of 24 countries (Denmark and Russia were excluded because of zero counts in the top level of variable V49 (frequency of prayer)). In this table, “config” refers to a configural model (thresholds *ν*_*g*_, loadings Λ_*g*_ and intercepts *τ*_*g*_ free across the groups); “metric” refers to a metric-invariant model (thresholds *ν*_*g*_ and loadings Λ_*g*_ constrained to be equal across groups; intercepts *τ*_*g*_ free across the groups); “scalar” refers to a scalar-invariant model (thresholds *ν*_*g*_, loadings Λ_*g*_ and intercepts *τ*_*g*_ constrained to be equal across the groups); “scalar partial” refers to a scalar-invariant model in which some of the constraints on the intercepts were released; and “strict” refers to a model in which the thresholds *ν*_*g*_, loadings Λ_*g*_, intercepts *τ*_*g*_ and residual variances Θ_*g*_ were constrained to be equal across the groups.

Grouping	Model	*χ*^2^	*df*	$\frac{\chi^{2}}{df}$	*p-value*	Δ*χ*^2^	Δ*df*	Pr(>*χ*^2^)	CFI	RMSEA (90% c.i.)	SRMR
SEX	config	1436.9	62	23	< 0.001	–	–	–	0.999	0.041 (0.040,0.043)	0.018
	metric	1459.5	83	18	< 0.001	64.4	21	< 0.001	0.999	0.036 (0.034,0.037)	0.018
	scalar	1701.2	90	19	< 0.001	434.1	7	< 0.001	0.999	0.037 (0.036,0.039)	0.018
	strict	1881.2	100	19	< 0.001	114.4	10	< 0.001	0.999	0.037 (0.036,0.039)	0.019
AGE	config	1422.4	155	9	< 0.001	–	–	–	0.999	0.040 (0.038,0.042)	0.018
	metric	1615.9	239	7	< 0.001	439.1	84	< 0.001	0.999	0.033 (0.032,0.035)	0.018
	scalar	2074.4	267	8	< 0.001	622.4	28	< 0.001	0.999	0.036 (0.035,0.038)	0.018
	strict	2562.2	307	8	< 0.001	234.9	40	< 0.001	0.999	0.038 (0.036,0.039)	0.021
DEGREE	config	1571	186	8	< 0.001	–	–	–	0.999	0.042 (0.040,0.043)	0.019
	metric	1851.8	291	6	< 0.001	626.6	105	< 0.001	0.999	0.035 (0.034,0.037)	0.019
	scalar	2382.6	326	7	< 0.001	706	35	< 0.001	0.999	0.038 (0.037,0.040)	0.019
	strict	3180.8	376	8	< 0.001	412.2	50	< 0.001	0.999	0.042 (0.040,0.043)	0.022
RELIGGRP	config	1798.9	155	12	< 0.001	–	–	–	0.999	0.045 (0.043,0.047)	0.026
	metric	3249	239	14	< 0.001	2518.5	84	< 0.001	0.998	0.049 (0.048,0.051)	0.027
	scalar	4908.9	267	18	< 0.001	1532	28	< 0.001	0.996	0.058 (0.057,0.059)	0.03
	strict	6967.1	307	23	< 0.001	1124.8	40	< 0.001	0.995	0.065 (0.063,0.066)	0.038
COUNTRY	config	2679.9	744	4	< 0.001	–	–	–	0.999	0.051 (0.049, 0.053)	0.031
	metric	6047.1	1227	5	< 0.001	5671.8	483	< 0.001	0.998	0.062 (0.061,0.064)	0.033
	scalar	10512.9	1388	8	< 0.001	4829.1	161	< 0.001	0.996	0.081 (0.079,0.082)	0.034
	strict	21873	1618	14	< 0.001	5744.9	230	< 0.001	0.991	0.112 (0.110,0.113)	0.061
COUNTRY*	config	2605.3	713	4	< 0.001	–	–	–	0.999	0.051 (0.049,0.053)	0.031
	metric	5326.4	1175	5	< 0.001	4560.6	462	< 0.001	0.998	0.059 (0.058,0.061)	0.033
	scalar	9577	1329	7	< 0.001	4689.9	154	< 0.001	0.996	0.078 (0.077,0.080)	0.033
	strict	17030.8	1549	11	< 0.001	4032.9	220	< 0.001	0.992	0.100 (0.098,0.101)	0.051
COUNTRY**	config	1501	434	3	< 0.001	–	–	–	0.999	0.051 (0.048,0.054)	0.027
	metric	2696.9	707	4	< 0.001	2222.4	273	< 0.001	0.999	0.055 (0.053,0.057)	0.028
	scalar	3249	759	4	< 0.001	780.7	52	< 0.001	0.998	0.059 (0.057,0.061)	0.029
	partial										

* The Netherlands was excluded.

** This model includes the following countries: Austria, Australia, Czech Republic, Hungary, Italy, Latvia, New Zealand, Norway, Poland, Portugal, Slovak Republic, Slovenia, United Kingdom and the United States. The intercepts *τ*_33_, *τ*_35_ and *τ*_*ATTEND*_ were freed across countries.

To improve this latter model, we had to use the modification indices to identify which intercepts should be freed in each factor for the best overall fit improvement and to inspect the countries’ *χ*^2^ contributions. First, we freed one intercept in each factor to see if we could obtain partial scalar invariance. Since this was not attained, we sequentially removed the countries until we obtained a model with acceptable fit. In this way, we obtained partial scalar invariance for 14 countries (see Table 4). In summary, the results of the tests for scalar invariance across religious (un)affiliation groups and countries only provided evidence for accepting the hypothesis of partial scalar invariance of the three-factor Model 2, and for a subset of 14 Christian-traditional countries included in the ISSP Religion Cumulation dataset.

We will now present some results on structural invariance. Table 5 shows the results of the structural invariance tests for SEX (gender), AGE (age group), DEGREE (highest educational degree) and RELIGGRP (religious group). We decided that structural invariance tests across the countries were not necessary. The hypothesis of invariant factor variance-covariance across groups was rejected for the religious (un)affiliation groups. Although we were not able to obtain scalar invariance across all grouping variables, the results in Table 5 suggest that if the group mean structures can be meaningfully compared, they should be different for all grouping variables considered. This is in agreement with many existing empirical studies, as discussed in the next section.

**Table 5:** Model 2: Structural invariance tests. Estimates of the GOF indices for the structural invariance (group variance-covariance and latent means) tests for model 2, for gender, age group, highest educational degree, and religious (un)affiliation. In this table, “metric” refers to a metric-invariant model (thresholds *ν*_*g*_ and loadings Λ_*g*_ constrained to be equal across groups; intercepts *τ*_*g*_ free across groups); “var.cov” refers to a metric-invariant model in which the variance-covariance matrices of the latent variables Φ_*g*_ are also constrained to be equal across the groups; “scalar” refers to a scalar-invariant model (thresholds *ν*_*g*_, loadings Λ_*g*_ and intercepts *τ*_*g*_ constrained to be equal across the groups); and “means” refers to a scalar-invariant model in which the latent means *κ*_*g*_ were also constrained to be equal across the groups.

Grouping	Model	*χ*^2^	*df*	$\frac{\chi^{2}}{df}$	*p-value*	Δ*χ*^2^	Δ*df*	Pr(>*χ*^2^)	CFI	RMSEA (90% c.i.)	SRMR
SEX	metric	1459.5	83	18	< 0.001	–	–	–	0.999	0.036 (0.034,0.037)	0.018
	var.cov	1738.2	89	20	< 0.001	75.2	6	< 0.001	0.999	0.038 (0.036,0.039)	0.019
	scalar	1701.2	90	19	< 0.001	–	–	–	0.999	0.037 (0.036,0.039)	0.018
	means	12757.4	93	137	< 0.001	478.2	3	< 0.001	0.994	0.103 (0.101,0.104)	0.019
AGE	metric	1615.9	239	7	< 0.001	–	–	–	0.999	0.033 (0.032,0.035)	0.018
	var.cov	2278	263	9	< 0.001	147.6	24	< 0.001	0.999	0.039 (0.037,0.040)	0.019
	scalar	2074.4	267	8	< 0.001	–	–	–	0.999	0.036 (0.035,0.038)	0.018
	means	9806.3	279	35	< 0.001	274.6	12	< 0.001	0.995	0.081 (0.080,0.083)	0.018
DEGREE	metric	1851.8	291	6	< 0.001	–	–	–	0.999	0.035 (0.034,0.037)	0.019
	var.cov	2835.7	321	9	< 0.001	210.4	30	< 0.001	0.999	0.043 (0.041,0.044)	0.023
	scalar	2382.6	326	7	< 0.001	–	–	–	0.999	0.038 (0.037,0.040)	0.019
	means	11184.7	341	33	< 0.001	325.1	15	< 0.001	0.995	0.086 (0.085,0.087)	0.019
RELIGGRP*	metric	3249	239	14	< 0.001	–	–	–	0.998	0.049 (0.049,0.051)	0.027
	var.cov	6124.4	263	23	< 0.001	543.1	24	< 0.001	0.995	0.066 (0.064,0.067)	0.033

* The model with constrained group means did not converge.

## Discussion

In this section, we will discuss the results presented above by first considering dimensionality (related to our first research question) and then measurement invariance (related to our second research question).

### Dimensionality

The results for the best fitting model suggested by the EFA and tested using MGCFA showed that three “core” dimensions of religiosity could be extracted from the ISSP Religion Cumulation dataset for historically Christian countries. These dimensions are represented by three factors that can be related to dimensions found in previous theoretical and empirical studies on the dimensions of religiosity [19, 25, 26, 28], but that association is not equally clear for all the factors.

Our factor “Beliefs in afterlife and miracles” is measured by four items that closely match corresponding items in the SBS-6 [21], and have particular significance within the official doctrine of Christian religion [63]. This factor’s structure came out identical in the two models obtained in the EFA. All the items in this factor have high communality, and the remaining items (which load on the other two factors) have weak loadings on it. Thus, this factor has a clear meaning and its measurement model is well defined by the four items V30-V33 in the ISSP Religion Cumulation dataset.

The association of the other two factors (“Belief and importance of God” and “Religious involvement”) with previous literature is not as clear as for “Beliefs in afterlife and miracles” and illustrates some of the limitations of the ISSP Religion Cumulation dataset. In particular, it is important to explain the cross-loading of our transformed item V28 (related to the level of belief in God) on two factors of apparently distinct nature (one related to believing and the other to behaving). Recall that the ten-item EFA solution showed that the variance of this item is nearly half spread between these two factors and that the MGCFA models for testing the measurement invariance across the religious groups and countries had improved fit when this item loaded on both factors.

Our factor “Belief and importance of God” associates belief in God with God’s role as a supernatural agent that cares and provides meaning to the life of every human being. A factor with this interpretation can be associated with the (considerably more complicated) “Belief” dimension in Glock and Stark’s religiosity scale [25, 48] and with the “Believing” dimension of the 4-BDRS [28]. However, as pointed out by Argyle [61] and other authors [101, 102], individuals hold different images of God. Some people believe in a personal God while others view God as a more abstract power, spirit or life-force [61]. Consequently, our factor may have a universal meaning, with those believing in a personal God scoring higher on it. Although other factors related to God may exist, we cannot detect them using the items in the ISSP Religion Cumulation dataset. To determine whether there is more than one factor related to God, it would be necessary to avoid coding belief in God in categories that confound level and meaning (as is the case of V28 in the ISSP Religion questionnaire and item 3 in Glock and Stark’s “Belief dimension” [25, 48]) and to include other items for better tapping “God” as a potentially multidimensional construct.

As we mentioned above in the analysis of the EFA, three of the items loading on the “Religious involvement” factor we found are closely associated with the three items in the “Religious involvement” factor found by Meuleman and Billiet [19] in a cross-cultural study based on the ESS. However, since our item V49 (frequency of prayer) does not differentiate between private and ritual prayer (at regular church services) our measurement of this factor is necessarily less precise.

The finding that item V28 also loads on the “religious involvement” factor and thus is cross-loading is perhaps our most intriguing result. It is worthwhile noting that this is not in contradiction with the findings of Meuleman and Billiet [19], because the ESS does not include items for measuring religious beliefs. We found that this cross-loading improved the fit of the MGCFA models for the religious groups and countries, but it is necessary to discuss the theoretical consistency of this result.

The findings on the psychological nature of religious beliefs indeed provide a plausible explanation of the direct influence of belief in God on “Religious involvement” (or “commitment”). Argyle [61] points out that religious beliefs are different from other beliefs in that they not consist of subjective agreement with verbal propositions, and combine emotions, personal attachments and commitment to action. In this author’s words, “We have emphasised that religious beliefs are different from believing, for example, in Julius Cesar, in that religious beliefs are combined with emotions, personal attachments and commitment to action—indeed, commitment to a whole way of life. Perhaps the most important difference is in the commitment to action, the implications of religious belief for moral behaviour”, and “Church attendance may be expected by members of a church, and those who do not attend are not regarded as proper believers.” ([61], pages 94-96). These theoretical arguments lend some support to our empirical finding of belief in God also measuring religious involvement.

In summary, we were able to extract three “core” dimensions of religiosity out of the ISSP Religion Cumulation dataset but with one critical item (related to the level of belief in God) cross-loading on two factors. Although this situation should be avoided when constructing multi-dimensional scales, the cross-loading of item V28 in our models is likely an inescapable consequence of the fact that belief(s) in God may influence more than one dimension of religiosity.

### Measurement and structural invariance

We begin with a note on the patterns of variation of the fit measures in the measurement and structural invariance tests and then proceed with a more specific discussion of the results.

First, all *χ*^2^ and scaled Δ*χ*^2^ tests were significant (Tables 4 and S5–S8 in the Supporting Information). Following the recommendations of several previous studies involving large samples and number of groups, we did not rely on the *χ*^2^ and scaled Δ*χ*^2^ tests for accepting or rejecting the models. The *χ*^2^/*df* estimates are outside the generally considered range for acceptable fit [93], but this fit measure also appears to be unreliable for evaluating the models’ fit in our conditions, because it tended to improve for the models with greater complexity in which other indices revealed stronger misfit. The CFI and ΔCFI were generally within the cutoffs for accepting invariance. The RMSEA is the fit measure that provided the best discrimination between different models. It was also more sensitive than the CFI and ΔCFI to the imposition and releasing of invariance constraints and to the removal of groups (countries).

Regarding measurement invariance, we found evidence that the three factors are metric-invariant, for the MGCFA models with thresholds and loadings invariant across groups met our acceptance criteria when tested across gender, age group, educational degree and religious (un)affiliation and, with the exception of The Netherlands, also across the countries.

According to our criteria, the hypothesis of scalar invariance was accepted in the tests across gender, age, educational degree and religious (un)affiliation group, but rejected in the test with the MGCFA model across countries. Since in the latter model we had to free three intercepts and eliminate nine more countries to get acceptable fit measures, we can only claim that we found some evidence for partial scalar invariance of the factor model. This outcome is not surprising, given the difficulty of proving factorial scalar invariance across many different countries in large surveys [19, 103]. More research is required to clarify this issue, once the results of further rounds of the ISSP Religion questionnaire become available.

Scalar invariance allows for meaningful comparisons of the estimated latent means between different groups. Some authors argue that such comparisons are still meaningful with partial scalar invariance [14, 100], while others argue that this may lead to wrong conclusions when testing for the significance of mean differences across groups (see e.g. [104]). Here, we will follow the former viewpoint and show plots of the factors’ latent means across the sociodemographic variables considered, which will allow some interesting comparisons with previous studies.

Before proceeding, we need to note that measurement invariance cannot be understood in terms of fit measures and cutoffs only, especially in the context of studies involving large samples and many groups, so that we have to discuss the practical consequences of non-invariance. In our study, there are plausible explanations for the non-invariant intercepts: in the case of item V33 (“Do you believe in religious miracles?”) perhaps because miracles are no longer taken as plausible by many people [105]; in the case of item V35 (“Agree/Disagree: There is a God who concerns Himself with every human being personally”) by the fact that not all people view God as a personal care-providing supernatural agent [61, 101, 102]; and in the case of frequency of church attendance (ATTEND) because this is probably influenced by country-specific factors extraneous to religion [106, 107].

#### Gender

**Fig 5:**
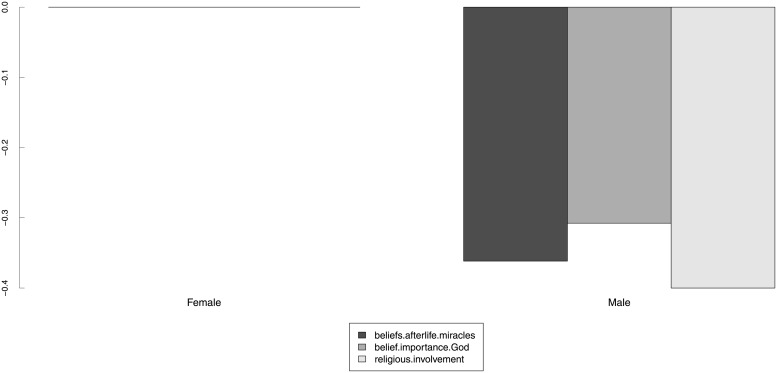
Latent means by gender. Group latent variable means (*κ*_*g*_) by gender, based on the model with scalar invariance (invariant thresholds, loadings and intercepts).

Fig 5 shows the latent means for the four factors by gender, based on the model with invariant *ν*, **Λ** and *τ* across these two groups. The differences between the latent means of the religiosity factors for the two sexes are consistent with the existing empirical evidence that women are more religious than men, at least in the context of Christian religion [38, 108–112].

#### Age

**Fig 6:**
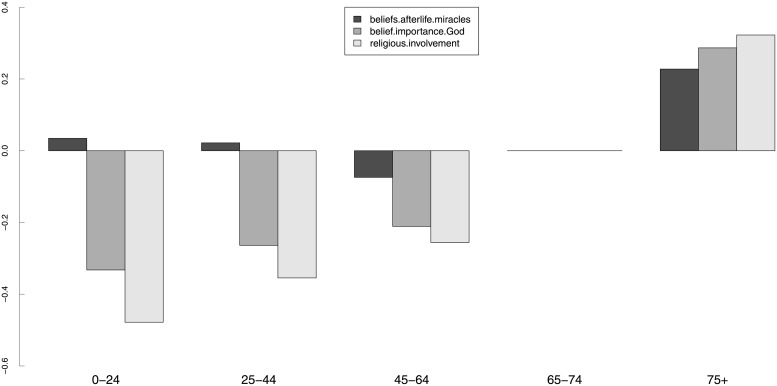
Latent means by age group. Group latent variable means (*κ*_*g*_) by age group, based on the model with scalar invariance (invariant thresholds, loadings and intercepts).

Fig 6 shows the latent means for the three factors by age group, based on the corresponding model with invariant *ν*, **Λ** and *τ*. The latent means of “Belief and importance of God” and “Religious involvement” increase monotonically with the age group (younger generations score lowest on these two factors), but the variation of “Beliefs in afterlife and miracles” with age has a “U” shape. Greeley [113] showed a “U” curve relationship between belief in life after death and age for East Germany and Russia (two countries that were under communist regimes for decades), based on the 1991 ISSP Religion data ([113], Fig 1). He claimed that a similar relationship was found in several other countries, but that the phenomenon of the younger being more religious than older is rarely observed. However, the results in Fig 6 suggest that the “U” curve variation of the level of afterlife beliefs is more general. This lends support to thanatocentric theories, which relate death anxiety to religious belief [21]. For example, it is known that fear of death increases in children and adolescents and decreases in adulthood [21, 114].

#### Educational degree

**Fig 7:**
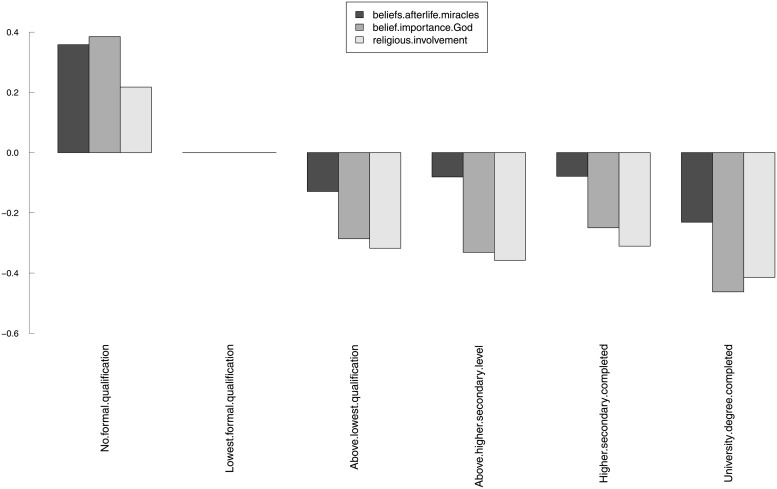
Latent means by highest educational degree. Group latent variable means (*κ*_*g*_) by highest educational degree, based on the model with scalar invariance (invariant thresholds, loadings and intercepts).

Fig 7 shows the latent means for the three factors by highest educational degree, based on the corresponding model with invariant *ν*, **Λ** and *τ*. This suggests the existence of salient differences between respondents with no and lowest formal qualification, and those with qualifications above lowest. There is mixed evidence in the literature on the relationship between education and religion [19, 115]. Some studies suggest a positive relationship [106, 116, 117], whereas others lean towards the opposite conclusion [115, 118, 119]. Our results clearly support the latter claim. Scholars of religion have expressed concern that psychological measurements relying on samples of University students are inappropriately narrow [120]. Our results also suggest that analyses of religiosity based on samples of University students may yield biased results, and that countries increasing their minimum qualification levels may lead to a decrease of the average level of religiosity (as argued by Hungerman [115] in relation to increasing compulsory schooling on the decline of religious affiliation in Canada).

#### Religious affiliation

**Fig 8:**
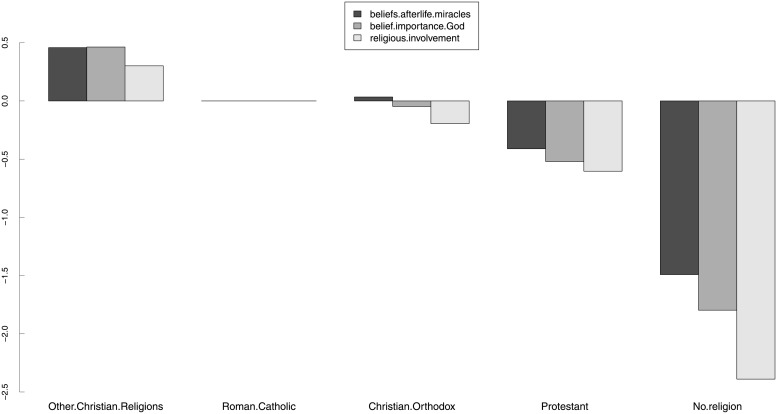
Latent means by religious (un)affiliation. Group latent variable means (*κ*_*g*_) by religious (un)affiliation, based on the model with scalar invariance (invariant thresholds, loadings and intercepts).

Fig 8 shows the latent means of the religiosity factors for the three Christian groups and the religiously unaffiliated, based on the corresponding model with invariant *ν*, **Λ** and *τ*. Three features of this figure are worthwhile noticing. First, respondents affiliated to ‘Other Christian Religions’ have the highest latent means of the three religiosity factors. This is consistent with earlier research on new religious movements showing that these groups often inspire high levels of commitment [121, 122]. Second, the ‘Protestant’ seem to be the least religious of the Christian groups. This is consistent with the well-known fact that Scandinavian countries, which still have high shares of Protestant-affiliated people, rank among the countries with lowest average levels of religious beliefs and involvement [123, 124]. Finally, the graphs in Fig 8 show that the religiously unaffiliated have much lower latent means of the three religiosity factors than all the Christian groups.

**Fig 9:**
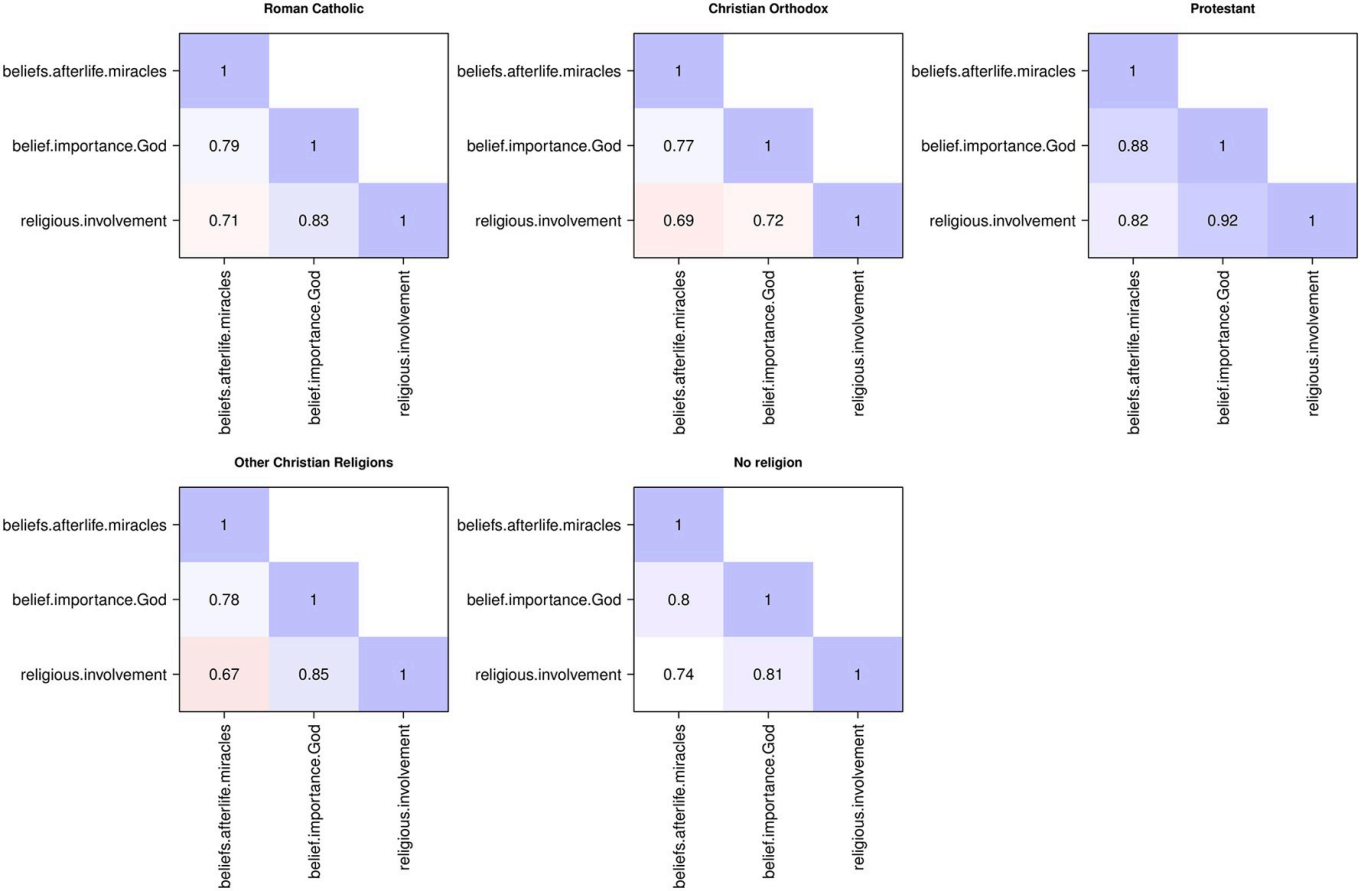
Factor correlation matrices for the Christian and ‘No religion’ groups. Factor correlation matrices for Christian and ‘No religion’ groups, based on the model with scalar invariance (invariant thresholds, loadings and intercepts).

It is also interesting to analyze the factor correlation structure for the religious (un)affiliation groups because the measurement and structural invariance tests provided evidence that the factors are common to all groups, but the groups’ factor variance-covariance matrices differ. Fig 9 shows graphical representations of the factor correlation matrices for the four Christian groups and for the unaffiliated. For all groups (Christian and religiously unaffiliated) all the correlations between factors are strong, and the correlation between “Beliefs in afterlife and miracles” and “Religious involvement” is the weakest inter-factor correlation. This is consistent with the factor solution shown in Fig 3.

#### Country

**Fig 10:**
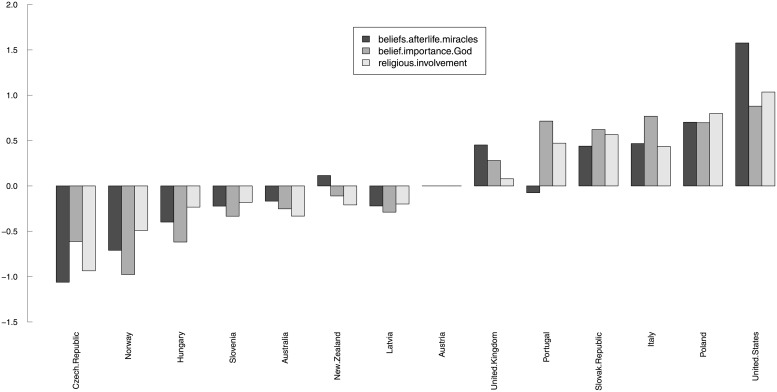
Latent means by country. Group latent variable means (*κ*_*g*_) by country for the 14 countries included in Model 2 with partial scalar invariance which led to acceptable fit (*τ*_33_, *τ*_35_ and *τ*_*ATTEND*_ free across countries).

Fig 10 shows the latent means of the religiosity factors for the fourteen countries in Model 2 with partial scalar invariance (Table 4). Although many countries had to be removed to obtain acceptable fit, particularly some highly religious countries like Ireland and the Philippines, this figure still demonstrates the diversity of religiosity across the Christian-traditional countries represented in the ISSP. On the left, highly secular countries with large shares of religiously unaffiliated persons (and Protestants in the case of Norway) have low latent means on the three factors. On the right, the four countries with strong Roman Catholic tradition (Portugal, Slovak Republic, Italy and Poland) have high latent means on the three factors. The United States also shows as a highly religious country, with latent mean of “Belief in afterlife and miracles” above the four Roman Catholic countries mentioned before. It is also interesting to notice that the latent means of “Belief in afterlife and miracles” are substantially different between Australia and New Zealand, which are countries with similar tradition.

The ordering of the countries’ religiosity suggested by Fig 10 closely matches the one shown in our qualitative analysis using faces plots (S3 Fig). It is also interesting to compare the latent means of religious involvement found in the present work with those reported by Meuleman and Billiet using the ESS Round 2, for the countries common to Fig 10 and Fig 7.2 in [19]: Czech Republic, Norway, Hungary, Slovenia, Austria, United Kingdom, Switzerland, Portugal, Slovak Republic and Poland, by increasing rank of latent means of “Religious involvement” in our solution. Except for swapping Slovenia and the United Kingdom, our ranks agree with those reported by Meuleman and Billiet [19].

### Limitations of the present study

The main limitations of the present work are due to two main aspects, the structure of the ISSP Religion Cumulation dataset and the criteria for rejection of the measurement invariance hypotheses in the MGCFA analyses.

The first limitation of the ISSP Religion Cumulation dataset results from the fact that it contains relatively few items that we could associate with those proposed in the main theories on religiosity, which significantly restricted the process of initial item selection. It should be noted that it would be virtually impossible to conceive and implement a multinational survey encompassing all the dimensions proposed in the major theories on religiosity. Consequently, the dimensions that can be studied using these surveys are limited by the items they include, so that important dimensions may remain hidden from the analysis. Such a problem may also arise in attempts to use other multinational datasets for studying the dimensionality of social constructs (religiosity, national identity, etc.). In the specific case of the ISSP Religion Cumulation dataset, there are critical items whose levels do not always have a clearly ordinal meaning (V28, related to belief in God), were asked in a way that does not differentiate contexts (V49, frequency of prayer) or were coded in too many levels (frequency of church attendance). This led to the need for collapsing levels, which resulted in potential degradation of the correlation structure. The items in these databases cannot, of course, be removed, added or revised a posteriori in the same way that the authors of religiosity scales have done to improve item communality, avoid cross-loadings and improve the scales’ reliability.

Another limitation of the present work is the use of the empirical criteria for rejecting invariance hypotheses in the MGCFA models, which are based on fixed cutoff values of the fit measures and their differences between successive nested models. In the case of the configural model the EFA helped providing confirmation of the model’s structure, but this was of course not possible for confirming the results on metric and (partial)scalar invariance. Although we followed conservative criteria proposed for WLSMV estimation of models with ordinal items, recent studies suggest that the use of permutation tests can improve control over Type I errors [125, 126]. However, these studies are also based on models with smaller samples and fewer groups than the ones considered herein and the permutation tests require a large number of simulations. The tentative application of these methods to our problem requires powerful computational resources and is left for further work. Approximate measurement invariance is another recent alternative [127]. This technique does not rely on exact constraints and is especially useful when the sample size is large and there are many small differences in the model parameters across the groups. This possibility is also left for further work.

## Conclusion

We studied dimensionality and factorial invariance of religiosity factors for Christian and religiously unaffiliated respondents from 26 historically Christian countries using the ISSP Religion Cumulation dataset. The study was performed in three stages. First, we selected an initial set of items from the ISSP Religion Cumulation dataset by combining elements from previous theoretical and empirical studies on dimensions of religiosity. Then, we used EFA to confirm the expected number of dimensions and identify alternative measurement models for the resulting factors. Finally, we tested different measurement models suggested by the EFA for factorial and structural invariance using MGCFA.

The results of the EFA indicated the presence of three factors, but the factor solutions showed items with borderline insufficient communality and cross-loading items. Furthermore, the EFA was not entirely conclusive about the measurement models (and consequently the theoretical interpretation) of the second and third factors. Thus, we studied four different three-factor models for configural, metric and scalar invariance using MGCFA.

The best fitting model tested using MGCFA is the one suggested by the best fitting solution obtained in the EFA and also the soundest from the viewpoint of theoretical interpretation. That model has three core factors of religiosity which we labeled “Beliefs in afterlife and miracles”, “Belief and importance of God” and “Religious involvement.” The first of these factors is measured by four items that closely correspond to items included in the SBS-6 scale [21]. The “Belief and importance of God” factor is measured by three items, one related to the level of belief in God and two to the psychological engagement with God as a supernatural agent (which concerns himself with every human being and gives life a meaning). A factor with this interpretation can be related to factors proposed in the Glock and Stark and 4-BDRS scales [25, 28]. The “Religious involvement” factor is measured by “self-rated religiosity” (or self-image as a religious person), the frequencies of prayer and church attendance and also by the level of belief in God that also loads on the “Belief and importance of God” factor. The first three of these items are related to the religious involvement factor reported by Meuleman and Billiet [19, 49], but because the ISSP item does not differentiate between private and public (ritual) prayer our measurement model for this factor is not as accurate as the one obtained from the ESS. The cross-loading of the item on belief in God may result either from the need to merge the levels of this item (to obtain a clearly ordinal transformed variable) or from belief in God effectively influencing religious involvement. Clarifying this doubt is an interesting topic for further research.

Previous theoretical and empirical studies had suggested the existence of dimensions similar to these factors. However, to the best of our knowledge, ours is the first study involving both Christian-affiliated and religiously unaffiliated individuals across sociodemographic groups and countries (ranging from highly secular to very religious) using a large dataset and testing the factors for measurement and structural invariance.

Regarding measurement invariance, we found evidence of metric invariance for the best-fitting three-factor model described above, based on tests across age, educational degree, religious (un)affiliation) and 23 countries (The Netherlands was excluded to meet the criteria for accepting metric invariance). In addition, the best-fitting MGCFA model with thresholds, loadings and intercepts invariant across groups led to acceptable fit measures and differences relative to the metric-invariant model when tested across sex, age, highest educational degree and religious (un)affiliation groups, but to poor fit measures when tested across the countries. In the latter model, we had to free one intercept on each factor and remove nine more countries (apart from The Netherlands) to improve the model’s fit and obtain acceptable fit measures. This provided some evidence for partial scalar invariance of the three factor model.

The idea of finding universal dimensions of social constructs and studying their measurement invariance using large multinational and cross-cultural datasets is appealing, because the scope, sample size, and diversity of these datasets can hardly be matched. The present work conveys a sense of the prospects and difficulties awaiting researchers that wish to pursue this idea.

## Supporting information

S1 File R script for importing a data frame with the ZA5070_v1-0-0 ISSP religion Cumulation data, select the Christian and religiously unaffiliated respondents and exclude the remaining respondents from Israel and Japan. (R)

S2 File R script for transforming items prior to the EFA and MGCFA. (R)

S1 Table R functions used for data inspection, exploratory factor analysis and confirmatory factor analysis. (PDF)

S2 Table Number of countries and respondents and % of respondents of each religious affiliation in the 1991, 1998 and 2008 rounds of the ISSP religion questionnaire. (PDF)

S3 Table GOF measures. Description of the GOF measures shown in the tables of multi-group measurement and structural invariance analyses, ranges for good and acceptable fit, and maximum differences of the fit measures for invariance in consecutive nested models.
(PDF)

S4 Table Map of items to face features in Chernoff faces plots. (PDF)

S5 Table Model 1: Fit measures for the measurement invariance tests. In this table, “config” refers to a configural model (thresholds *ν*_*g*_, loadings Λ_*g*_ and intercepts *τ*_*g*_ free across the groups); “metric” refers to a metric-invariant model (thresholds *ν*_*g*_ and loadings Λ_*g*_ constrained to be equal across groups; intercepts *τ*_*g*_ free across the groups); “scalar” refers to a scalar-invariant model (thresholds *ν*_*g*_, loadings Λ_*g*_ and intercepts *τ*_*g*_ constrained to be equal across the groups); and “strict” refers to a model in which the thresholds *ν*_*g*_, loadings Λ_*g*_, intercepts *τ*_*g*_ and residual variances Θ_*g*_ were constrained to be equal across the groups.
(PDF)

S6 Table Model 2: Fit measures for the measurement invariance tests. In this table, “config” refers to a configural model (thresholds *ν*_*g*_, loadings Λ_*g*_ and intercepts *τ*_*g*_ free across the groups); “metric” refers to a metric-invariant model (thresholds *ν*_*g*_ and loadings Λ_*g*_ constrained to be equal across groups; intercepts *τ*_*g*_ free across the groups); “scalar” refers to a scalar-invariant model (thresholds *ν*_*g*_, loadings Λ_*g*_ and intercepts *τ*_*g*_ constrained to be equal across the groups); and “strict” refers to a model in which the thresholds *ν*_*g*_, loadings Λ_*g*_, intercepts *τ*_*g*_ and residual variances Θ_*g*_ were constrained to be equal across the groups.
(PDF)

S7 Table Model 3: Fit measures for the measurement invariance tests. In this table, “config” refers to a configural model (thresholds *ν*_*g*_, loadings Λ_*g*_ and intercepts *τ*_*g*_ free across the groups); “metric” refers to a metric-invariant model (thresholds *ν*_*g*_ and loadings Λ_*g*_ constrained to be equal across groups; intercepts *τ*_*g*_ free across the groups); “scalar” refers to a scalar-invariant model (thresholds *ν*_*g*_, loadings Λ_*g*_ and intercepts *τ*_*g*_ constrained to be equal across the groups); and “strict” refers to a model in which the thresholds *ν*_*g*_, loadings Λ_*g*_, intercepts *τ*_*g*_ and residual variances Θ_*g*_ were constrained to be equal across the groups.
(PDF)

S8 Table Model 4: Fit measures for the measurement invariance tests. In this table, “config” refers to a configural model (thresholds *ν*_*g*_, loadings Λ_*g*_ and intercepts *τ*_*g*_ free across the groups); “metric” refers to a metric-invariant model (thresholds *ν*_*g*_ and loadings Λ_*g*_ constrained to be equal across groups; intercepts *τ*_*g*_ free across the groups); “scalar” refers to a scalar-invariant model (thresholds *ν*_*g*_, loadings Λ_*g*_ and intercepts *τ*_*g*_ constrained to be equal across the groups); and “strict” refers to a model in which the thresholds *ν*_*g*_, loadings Λ_*g*_, intercepts *τ*_*g*_ and residual variances Θ_*g*_ were constrained to be equal across the groups.
(PDF)

S9 Table Levels of measurement and structural invariance, models and invariance constraints. (PDF)

S1 Fig Missing data pattern. Missing data pattern of the selected items for year 1998 (based on [50]).
(EPS)

S2 Fig Chernoff faces by religious group and educational degree. Chernoff faces plot of the medians of the selected items shown in S4 Table for each combination of religious group and educational degree, for year 1998 (based on [50]).
(EPS)

S3 Fig Chernoff faces by country. Chernoff faces plot of the medians of the selected items shown in S4 Table for each country, for year 1998 (based on [50]).
(EPS)

S1 Appendix The linear common factor model. (PDF)
