# Supplementary material for: Dimensionality and factorial invariance of religiosity among Christians and the religiously unaffiliated: A cross-cultural analysis based on the International Social Survey Programme
Source: PLoS One. 2019 May 15;14(5):e0216352. doi: 10.1371/journal.pone.0216352 (PMC6519809; doi:10.1371/journal.pone.0216352)
Supplement: S1 Table — (PDF) [file pone.0216352.s003.pdf]

Function	Package	Usage
`aggr`	`VIM`	Display missing data patterns [57]
`cfa`	`lavaan`	Confirmatory factor analysis, constrained and unconstrained [55]
`cor.plot`	`psych`	Graphical representation of the structure of correlation matrices [56]
`fa`	`psych`	Computation of factor solutions in Exploratory Factor Analysis [56]
`faces`	`aplpack`	Graphical representation of Chernoff faces plots [54]
`fa.parallel`	`psych`	Determination of the number of factors to extract via a Monte Carlo simulation [56]
`fa.diagram`	`psych`	Graphical representation of factor solutions obtained using `fa` [56]
`lavInspect`	`lavaan`	Extract information from `lavaan` models [55]
`lavTestLRT`	`lavaan`	Computation of the likelihood ratio test of scaled χ^2 differences for comparing nested `lavaan` models [55]
`measEq.syntax`	`semTools`	Generates `lavaan` model syntax for CFA [58]; also computes CFA solutions using `lavaan` [55]
`read.dta`	`foreign`	Import Stata files into `R` [53]
`spineplot`	`graphics`	Display group contingency tables in exploratory data analysis [52]
`statsBy`	`psych`	Computation of the pooled within-group correlation matrices [56]
`vss`	`psych`	Determination of the number of factors to extract using the Very Simple Structure (VSS) and Velicer's Minimum Average Partial (MAP) criteria [56]
