# Supplementary material for: Dimensionality and factorial invariance of religiosity among Christians and the religiously unaffiliated: A cross-cultural analysis based on the International Social Survey Programme
Source: PLoS One. 2019 May 15;14(5):e0216352. doi: 10.1371/journal.pone.0216352 (PMC6519809; doi:10.1371/journal.pone.0216352)
Supplement: S2 Table — (PDF) [file pone.0216352.s004.pdf]

	1991	1998	2008
Number of countries in dataset	16	28	28
Number of respondents in dataset	24970	36958	40526
No Religion (%)	24.1	22.98	24.93
Roman Catholic (%)	38.75	41.14	40.6
Protestant (%)	26.99	22.62	21.12
Christian Orthodox (%)	3.76	5.72	5.26
Jewish (%)	4.14	3.06	2.73
Islam (%)	0.36	0.68	1.35
Buddhism (%)	0.04	1.17	1.16
Hinduism (%)	0.04	0.02	0.15
Other Christian Religions (%)	0.24	1.01	1.81
Other Eastern Religions (%)	0.01	0.07	0.12
Other Religions (%)	1.03	1.02	0.77
No (Christian) denomination given (%)	0.53	0.49	–

NOTE: The religious affiliations correspond to the categories of the `RELIGGRP` background variable in the ISSP Religion Cumulation dataset [50].
