# Supplementary material for: Dimensionality and factorial invariance of religiosity among Christians and the religiously unaffiliated: A cross-cultural analysis based on the International Social Survey Programme
Source: PLoS One. 2019 May 15;14(5):e0216352. doi: 10.1371/journal.pone.0216352 (PMC6519809; doi:10.1371/journal.pone.0216352)
Supplement: S3 Table — Description of the GOF measures shown in the tables of multi-group measurement and structural invariance analyses, ranges for good and acceptable fit, and maximum differences of the fit measures for invariance in consecutive nested models. (PDF) [file pone.0216352.s005.pdf]

Measure	Definition	Description	Criteria
χ^2 (df)	$(N-1)F(\mathbf{S}, \mathbf{\Sigma}(\hat{\Theta}))$	Test statistic based on the minimum of fit function	–
RMSEA	$\sqrt{max\{(\chi^2/\text{df}-1)/(N-1)-1,0\}}$	Root Mean Square of Approximation	RMSEA ≤ 0.05 good fit<br>$0.05 <$ RMSEA ≤ 0.06 acceptable fit
SRMR	$\sqrt{\sum_{i=1}^{p}\sum_{j=1}^{i}\frac{s_{ij}-\hat{\sigma}_{ij}}{s_i s_j}\frac{2}{p(p+1)}}$	Standardized Root Mean Square Residual	SRMR ≤ 0.05 good fit<br>$0.05 <$ SRMR ≤ 0.10 acceptable fit
CFI	$1-\frac{max[(\chi_t^2-\text{df}_t),0]}{max[(\chi_t^2-\text{df}_t),(\chi_i^2-\text{df}_i),0]}$	Comparative Fit Index	CFI ≥ 0.97 good fit<br>$0.95 \leq$ CFI < 0.97 acceptable fit
ΔCFI		Difference between CFI of consecutive nested models	Δ(CFI) > -0.002
ΔRMSEA		Difference between RMSEA of consecutive nested models	Δ(RMSEA) < 0.015
ΔSRMR		Difference between SRMR of consecutive nested models	Δ(SRMR) < 0.03 (metric invariance)<br>Δ(SRMR) < 0.01 (scalar/strict invariance)

Description of symbols: $\mathbf{S}$ is the empirical variance-covariance matrix; $\mathbf{\Sigma}(\hat{\Theta})$ is the model-implied variance-covariance matrix; $\hat{\Theta}$ is the vector of estimated model parameters; $F(\mathbf{S}, \mathbf{\Sigma}(\hat{\Theta}))$ is the fitting function (which depends on the discrepancy between empirical and model-implied variance-covariance matrices and the estimator used); s_{ij} and $\hat{\sigma_{ij}}$ are elements of $\mathbf{S}$ and $\mathbf{\Sigma}(\hat{\Theta})$ respectively, and $s_i = \hat{\sigma}_{ij}$; p is the number of observed items; χ_t^2, χ_i^2 and χ_n^2 are the χ^2 of the target, baseline and null models; and df, df_t, df_i and df_n are the numbers of degrees of freedom of the model, and target, baseline and null models [86], respectively.
