# Supplementary material for: Dimensionality and factorial invariance of religiosity among Christians and the religiously unaffiliated: A cross-cultural analysis based on the International Social Survey Programme
Source: PLoS One. 2019 May 15;14(5):e0216352. doi: 10.1371/journal.pone.0216352 (PMC6519809; doi:10.1371/journal.pone.0216352)
Supplement: S4 Table — (PDF) [file pone.0216352.s006.pdf]

**Feature**	**Item**	**Label**
Height of face	V49	About how often do you pray?
Width of face	ATTEND	How often do you attend religious services?
Structure of face	V51	Would you describe yourself as religious?
Height of mouth	V31	Do you believe in heaven?
Width of mouth	V32	Do you believe in hell?
Smiling	V31	Do you believe in heaven?
Height of eyes	V30	Do you believe in life after death?
Width of eyes	V30	Do you believe in life after death?
Height of hair	V35	Agree/Disagree: There is a God who concerns Himself with every human being personally.
Width of hair	V37	Agree/Disagree: To me, life is meaningful only because God exists.
Style of hair	V28	Please indicate which statement comes closest to expressing what you believe about God.
Height of nose	V33	Do you believe in religious miracles?
Width of nose	V33	Do you believe in religious miracles?
Width of ear	V50	How often do you take part in the activities of a church or place of worship other than attending services?
Height of ear	V50	How often do you take part in the activities of a church or place of worship other than attending services?
