# Supplementary material for: Dimensionality and factorial invariance of religiosity among Christians and the religiously unaffiliated: A cross-cultural analysis based on the International Social Survey Programme
Source: PLoS One. 2019 May 15;14(5):e0216352. doi: 10.1371/journal.pone.0216352 (PMC6519809; doi:10.1371/journal.pone.0216352)
Supplement: S5 Table — In this table, “config” refers to a configural model (thresholds νg, loadings Λg and intercepts τg free across the groups); “metric” refers to a metric-invariant model (thresholds νg and loadings Λg constrained to be equal across groups; intercepts τg free across the groups); “scalar” refers to a scalar-invariant model (thresholds νg, loadings Λg and intercepts τg constrained to be equal across the groups); and “strict” refers to a model in which the thresholds νg, loadings Λg, intercepts τg and residual variances Θg were constrained to be equal across the groups. (PDF) [file pone.0216352.s007.pdf]

**Grouping**	**Model**	χ^2	*df*	$\frac{\chi^2}{df}$	*p-value*	$\Delta\chi^2$	Δdf	Pr(> χ^2)	CFI	RMSEA (90% c.i.)	SRMR
SEX	config	2596.7	80	32	< 0.001	–	–	–	0.999	0.049 (0.048,0.051)	0.023
	metric	2628.1	103	26	< 0.001	81.0	23	< 0.001	0.999	0.044 (0.042,0.045)	0.023
	scalar	2986.0	111	27	< 0.001	528.6	8	< 0.001	0.999	0.045 (0.043,0.046)	0.023
	strict	3147.4	122	26	< 0.001	96.0	11	< 0.001	0.999	0.044 (0.043,0.045)	0.024
AGE	config	2525.9	200	13	< 0.001	–	–	–	0.999	0.048 (0.046,0.049)	0.023
	metric	2751.4	292	9	< 0.001	460.9	92	< 0.001	0.999	0.049 (0.039,0.042)	0.023
	scalar	3300.8	324	10	< 0.001	632.9	32	< 0.001	0.999	0.042 (0.041,0.044)	0.023
	strict	3754.7	368	10	< 0.001	204.9	44	< 0.001	0.998	0.042 (0.041,0.044)	0.025
DEGREE	config	2787.7	240	12	< 0.001	–	–	–	0.999	0.050 (0.048,0.051)	0.024
	metric	3062.6	355	9	< 0.001	556.5	115	< 0.001	0.999	0.042 (0.041,0.044)	0.024
	scalar	3724.8	395	9	< 0.001	749.7	40	< 0.001	0.998	0.044 (0.043,0.046)	0.024
	strict	4569.8	450	10	< 0.001	402.0	55	< 0.001	0.998	0.046 (0.045,0.047)	0.027
RELIGGRP	config	3054.9	200	15	< 0.001	–	–	–	0.998	0.053 (0.051,0.054)	0.032
	metric*	–	–	–	–	–	–	–	–	–	–
	scalar	7153.5	324	22	< 0.001	2301.3	32	< 0.001	0.995	0.064 (0.063,0.065)	0.035
	strict	9041.5	368	25	< 0.001	958.7	44	< 0.001	0.993	0.068 (0.066,0.069)	0.040
COUNTRY	config	4251.6	960	4	< 0.001	–	–	–	0.999	0.058 (0.057,0.060)	0.036
	metric*	–	–	–	–	–	–	–	–	–	–
	scalar*	–	–	–	–	–	–	–	–	–	–
	strict	26751.6	1926	14	< 0.001	5883.2	253	< 0.001	0.989	0.113 (0.112,0.115)	0.065

* Solution invalid due to negative model-implied item variances.
