# Supplementary material for: Dimensionality and factorial invariance of religiosity among Christians and the religiously unaffiliated: A cross-cultural analysis based on the International Social Survey Programme
Source: PLoS One. 2019 May 15;14(5):e0216352. doi: 10.1371/journal.pone.0216352 (PMC6519809; doi:10.1371/journal.pone.0216352)
Supplement: S6 Table — In this table, “config” refers to a configural model (thresholds νg, loadings Λg and intercepts τg free across the groups); “metric” refers to a metric-invariant model (thresholds νg and loadings Λg constrained to be equal across groups; intercepts τg free across the groups); “scalar” refers to a scalar-invariant model (thresholds νg, loadings Λg and intercepts τg constrained to be equal across the groups); and “strict” refers to a model in which the thresholds νg, loadings Λg, intercepts τg and residual variances Θg were constrained to be equal across the groups. (PDF) [file pone.0216352.s008.pdf]

**Grouping**	**Model**	χ^2	*df*	$\frac{\chi^2}{df}$	*p-value*	$\Delta\chi^2$	Δdf	Pr($> \chi^2$)	CFI	RMSEA (90% c.i.)	SRMR
SEX	config	1436.9	62	23	< 0.001	–	–	–	0.999	0.041 (0.040,0.043)	0.018
	metric	1459.5	83	18	< 0.001	64.4	21	< 0.001	0.999	0.036 (0.034,0.037)	0.018
	scalar	1701.2	90	19	< 0.001	434.1	7	< 0.001	0.999	0.037 (0.036,0.039)	0.018
	strict	1881.2	100	19	< 0.001	114.4	10	< 0.001	0.999	0.037 (0.036,0.039)	0.019
AGE	config	1422.4	155	9	< 0.001	–	–	–	0.999	0.040 (0.038,0.042)	0.018
	metric	1615.9	239	7	< 0.001	439.1	84	< 0.001	0.999	0.033 (0.032,0.035)	0.018
	scalar	2074.4	267	8	< 0.001	622.4	28	< 0.001	0.999	0.036 (0.035,0.038)	0.018
	strict	2562.2	307	8	< 0.001	234.9	40	< 0.001	0.999	0.038 (0.036,0.039)	0.021
DEGREE	config	1571.0	186	8	< 0.001	–	–	–	0.999	0.042 (0.040,0.043)	0.019
	metric	1851.8	291	6	< 0.001	626.6	105	< 0.001	0.999	0.035 (0.034,0.037)	0.019
	scalar	2382.6	326	7	< 0.001	706.0	35	< 0.001	0.999	0.038 (0.037,0.040)	0.019
	strict	3180.8	376	8	< 0.001	412.2	50	< 0.001	0.999	0.042 (0.040,0.043)	0.022
RELIGGRP	config	1798.9	155	12	< 0.001	–	–	–	0.999	0.045 (0.043,0.047)	0.026
	metric	3249.0	239	14	< 0.001	2518.5	84	< 0.001	0.998	0.049 (0.048,0.051)	0.027
	scalar	4908.9	267	18	< 0.001	1532.0	28	< 0.001	0.996	0.058 (0.057,0.059)	0.030
	strict	6967.1	307	23	< 0.001	1124.8	40	< 0.001	0.995	0.065 (0.063,0.066)	0.038
COUNTRY	config	2679.9	744	4	< 0.001	–	–	–	0.999	0.051 (0.049, 0.053)	0.031
	metric	6047.1	1227	5	< 0.001	5671.8	483	< 0.001	0.998	0.062 (0.061,0.064)	0.033
	scalar	10512.9	1388	8	< 0.001	4829.1	161	< 0.001	0.996	0.081 (0.079,0.082)	0.034
	strict*	–	–	–	–	–	–	–	–	–	–

* Solution invalid due to covariance matrix of latent variables not positive definite for Norway and Sweden.
