# Supplementary material for: Dimensionality and factorial invariance of religiosity among Christians and the religiously unaffiliated: A cross-cultural analysis based on the International Social Survey Programme
Source: PLoS One. 2019 May 15;14(5):e0216352. doi: 10.1371/journal.pone.0216352 (PMC6519809; doi:10.1371/journal.pone.0216352)
Supplement: S7 Table — In this table, “config” refers to a configural model (thresholds νg, loadings Λg and intercepts τg free across the groups); “metric” refers to a metric-invariant model (thresholds νg and loadings Λg constrained to be equal across groups; intercepts τg free across the groups); “scalar” refers to a scalar-invariant model (thresholds νg, loadings Λg and intercepts τg constrained to be equal across the groups); and “strict” refers to a model in which the thresholds νg, loadings Λg, intercepts τg and residual variances Θg were constrained to be equal across the groups. (PDF) [file pone.0216352.s009.pdf]

Grouping	Model	χ^2	*df*	$\frac{\chi^2}{df}$	*p-value*	$\Delta\chi^2$	Δdf	Pr(> χ^2)	CFI	RMSEA (90% c.i.)	SRMR
SEX	config	1816.6	64	28	< 0.001	–	–	–	0.999	0.046 (0.044,0.048)	0.020
	metric	1837.2	84	22	< 0.001	58.8	20	< 0.001	0.999	0.040 (0.039,0.042)	0.020
	scalar	2080.7	91	23	< 0.001	433.5	7	< 0.001	0.999	0.041 (0.040,0.043)	0.020
	strict	2258.4	101	22	< 0.001	113.1	10	< 0.001	0.999	0.041 (0.039,0.042)	0.020
AGE	config	1845.7	160	12	< 0.001	–	–	–	0.999	0.045 (0.043,0.047)	0.020
	metric	2039.7	240	8	< 0.001	437.9	80	< 0.001	0.999	0.038 (0.037,0.040)	0.020
	scalar	2482.2	268	9	< 0.001	596.7	28	< 0.001	0.999	0.040 (0.039,0.041)	0.020
	strict	2955.1	308	10	< 0.001	227.3	40	< 0.001	0.999	0.041 (0.039,0.042)	0.022
DEGREE	config	1984.3	192	10	< 0.001	–	–	–	0.999	0.047 (0.045,0.048)	0.021
	metric	2273.8	292	8	< 0.001	648.5	100	< 0.001	0.999	0.040 (0.038,0.041)	0.021
	scalar	2786.5	327	9	< 0.001	674.4	35	< 0.001	0.999	0.042 (0.040,0.043)	0.021
	strict	3563.3	377	9	< 0.001	399.8	50	< 0.001	0.998	0.044 (0.043,0.046)	0.023
RELIGGRP	config	2204.4	160	14	< 0.001	–	–	–	0.998	0.050 (0.048,0.052)	0.028
	metric	3699.7	240	15	< 0.001	2655.8	80	< 0.001	0.997	0.053 (0.051,0.054)	0.029
	scalar	5447.5	268	20	< 0.001	1603.8	28	< 0.001	0.996	0.061 (0.060,0.063)	0.031
	strict	7472.6	308	24	< 0.001	1101.4	40	< 0.001	0.994	0.067 (0.066,0.068)	0.040
COUNTRY	config	3142.4	768	4	< 0.001	–	–	–	0.999	0.055 (0.053,0.057)	0.032
	metric	6612.9	1228	5	< 0.001	5953.0	460	< 0.001	0.997	0.066 (0.064,0.068)	0.035
	scalar	11037.1	1389	8	< 0.001	4710.8	161	< 0.001	0.996	0.083 (0.082,0.085)	0.035
	strict*	–	–	–	–	–	–	–	–	–	–

* Solution invalid due to covariance matrix of latent variables not positive definite for Australia, Latvia, The Netherlands, New Zealand, Norway, Slovenia and Sweden.
