# Supplementary material for: Dimensionality and factorial invariance of religiosity among Christians and the religiously unaffiliated: A cross-cultural analysis based on the International Social Survey Programme
Source: PLoS One. 2019 May 15;14(5):e0216352. doi: 10.1371/journal.pone.0216352 (PMC6519809; doi:10.1371/journal.pone.0216352)
Supplement: S8 Table — In this table, “config” refers to a configural model (thresholds νg, loadings Λg and intercepts τg free across the groups); “metric” refers to a metric-invariant model (thresholds νg and loadings Λg constrained to be equal across groups; intercepts τg free across the groups); “scalar” refers to a scalar-invariant model (thresholds νg, loadings Λg and intercepts τg constrained to be equal across the groups); and “strict” refers to a model in which the thresholds νg, loadings Λg, intercepts τg and residual variances Θg were constrained to be equal across the groups. (PDF) [file pone.0216352.s010.pdf]

**Grouping**	**Model**	χ^2	*df*	$\frac{\chi^2}{df}$	*p-value*	$\Delta\chi^2$	Δdf	Pr($> \chi^2$)	CFI	RMSEA (90% c.i.)	SRMR
SEX	config	1949.1	64	30	< 0.001	–	–	–	0.999	0.048 (0.046,0.050)	0.022
	metric	1970.4	84	23	< 0.001	61.5	20	< 0.001	0.999	0.042 (0.040,0.043)	0.022
	scalar	2232.3	91	25	< 0.001	470.9	7	< 0.001	0.999	0.043 (0.041,0.044)	0.022
	strict	2416.1	101	24	< 0.001	117.3	10	< 0.001	0.999	0.042 (0.041,0.044)	0.022
AGE	config	1874.3	160	12	< 0.001	–	–	–	0.999	0.046 (0.044,0.047)	0.021
	metric	2057.1	240	9	< 0.001	422.3	80	< 0.001	0.999	0.038 (0.037,0.040)	0.021
	scalar	2541.5	268	9	< 0.001	653.7	28	< 0.001	0.999	0.041 (0.039,0.042)	0.021
	strict	3028.2	308	10	< 0.001	233.2	40	< 0.001	0.999	0.041 (0.040,0.043)	0.023
DEGREE	config	2068.9	192	11	< 0.001	–	–	–	0.999	0.048 (0.046,0.050)	0.022
	metric	2309.9	292	8	< 0.001	550.4	100	< 0.001	0.999	0.040 (0.039,0.042)	0.022
	scalar	2861.8	327	9	< 0.001	730.6	35	< 0.001	0.999	0.042 (0.041,0.044)	0.022
	strict	3687.3	377	10	< 0.001	425.1	50	< 0.001	0.998	0.045 (0.044,0.046)	0.025
RELIGGRP	config	2374.4	160	15	< 0.001	–	–	–	0.998	0.052 (0.050,0.054)	0.031
	metric	3750.5	240	16	< 0.001	2423.2	80	< 0.001	0.997	0.053 (0.052,0.055)	0.032
	scalar	5543.6	268	21	< 0.001	1637.3	28	< 0.001	0.996	0.062 (0.060,0.063)	0.034
	strict	7680.3	308	25	< 0.001	1139.7	40	< 0.001	0.994	0.068 (0.067,0.069)	0.043
COUNTRY	config	3122.1	768	4	< 0.001	–	–	–	0.999	0.055 (0.053,0.057)	0.034
	metric	6330.0	1228	5	< 0.001	5532.2	460	< 0.001	0.998	0.064 (0.063,0.066)	0.036
	scalar	10922.0	1389	8	< 0.001	4961.8	161	< 0.001	0.996	0.083 (0.081,0.084)	0.036
	strict*	–	–	–	–	–	–	–	–	–	–

* Solution invalid due to covariance matrix of latent variables not positive definite for Norway and Sweden.
