# Supplementary material for: Dimensionality and factorial invariance of religiosity among Christians and the religiously unaffiliated: A cross-cultural analysis based on the International Social Survey Programme
Source: PLoS One. 2019 May 15;14(5):e0216352. doi: 10.1371/journal.pone.0216352 (PMC6519809; doi:10.1371/journal.pone.0216352)
Supplement: S9 Table — (PDF) [file pone.0216352.s011.pdf]

Invariance level	Model	Invariance constraints
Configural		
Metric (weak)	Invariant ν, $\mathbf{\Lambda}$	$\nu_g = \nu$, $\mathbf{\Lambda}_g = \mathbf{\Lambda}$
Scalar (strong)	Invariant ν, $\mathbf{\Lambda}$, τ	$\nu_g = \nu$, $\mathbf{\Lambda}_g = \mathbf{\Lambda}$, $\tau_g = \tau$
Full (strict)	Invariant ν, $\mathbf{\Lambda}$, τ, $\mathbf{\Theta}$	$\nu_g = \nu$, $\mathbf{\Lambda}_g = \mathbf{\Lambda}$, $\tau_g = \tau$, $\mathbf{\Theta}_g = \mathbf{\Theta}$
Invariant $\mathbf{\Phi}$ *	Invariant ν, $\mathbf{\Lambda}$, $\mathbf{\Phi}$	$\nu_g = \nu$, $\mathbf{\Lambda}_g = \mathbf{\Lambda}$, $\mathbf{\Phi}_g = \mathbf{\Phi}$
Invariant κ **	Invariant ν, $\mathbf{\Lambda}$, τ, κ	$\nu_g = \nu$, $\mathbf{\Lambda}_g = \mathbf{\Lambda}$, $\tau_g = \tau$, $\kappa_g = \kappa$

Description of symbols: $\mathbf{\Lambda}$ is the matrix of factor loadings; ν is a vector or list of thresholds (this is represented by a matrix when all ordinal items have the same number of categories, as in e.g. [29,126]); τ is the vector of intercepts; $\mathbf{\Phi}$ is the variance-covariance matrix of the latent variables; $\mathbf{\Theta}$ is the variance-covariance matrix of the residuals (usually assumed diagonal); and the subscript g denotes the group.

* Requires metric invariance.

** Requires scalar invariance.
