# Supplementary material for: Dimensionality and factorial invariance of religiosity among Christians and the religiously unaffiliated: A cross-cultural analysis based on the International Social Survey Programme
Source: PLoS One. 2019 May 15;14(5):e0216352. doi: 10.1371/journal.pone.0216352 (PMC6519809; doi:10.1371/journal.pone.0216352)
Supplement: S1 Appendix — (PDF) [file pone.0216352.s015.pdf]

For continuous items (or indicators), the linear common factor model is:

$$\mathbf{X}_g = \tau_g + \mathbf{\Lambda}_g \xi_g + \delta_g, \tag{1}$$

where $\mathbf{X}_g$, τ_g and δ_g are the vectors of observed variables, intercepts and errors, respectively; $\mathbf{\Lambda}_g$ is the matrix of factor loadings; ξ_g is the vector of latent variables; and the subscript g denotes a group (or unit of analysis). It is assumed that $\xi_g \sim MVN(\kappa_g, \mathbf{\Phi}_g)$ and $\delta_g \sim MVN(\mathbf{0}_g, \mathbf{\Theta}_g)$ for each group. This implies that $\mathbf{X}_g \sim MVN(\mu_g, \mathbf{\Sigma}_g)$ and

$$\mu_g = \tau_g + \mathbf{\Lambda}_g \kappa_g, \tag{2}$$

$$\mathbf{\Sigma}_g = \mathbf{\Lambda}_g \mathbf{\Phi}_g \mathbf{\Lambda}_g' + \mathbf{\Theta}_g, \tag{3}$$

where $\mathbf{\Phi}_g$ is the variance-covariance matrix of the latent variables, and $\mathbf{\Theta}_g$ is the variance-covariance matrix of δ_g which is usually constrained to be diagonal [14]. For the model to be identified, the location and scale of the latent variables must be fixed. This can be done in different ways [14,16,81,84], for example by fixing the factors' means and variances, or setting one item loading to 1 and the corresponding intercept to 0 for each factor. The latter alternative is generally worse, because it may lead to problems if the chosen (marker) items are not invariant or the loading is much smaller than the other loadings in the same factor [83].

In the case of ordinal items, the observed scores $\mathbf{X}_g$ are assumed to be determined by unobserved latent response variables $\mathbf{X}_g^* \sim MVN(\mu_g^*, \mathbf{\Sigma}_g^*)$, and the factor model is

$$\mathbf{X}_g^* = \tau_g + \mathbf{\Lambda}_g \xi_g + \delta_g, \tag{4}$$

which implies that

$$\mu_g^* = \tau_g + \mathbf{\Lambda}_g \kappa_g, \tag{5}$$

$$\mathbf{\Sigma}_g^* = \mathbf{\Lambda}_g \mathbf{\Phi}_g \mathbf{\Lambda}_g' + \mathbf{\Theta}_g. \tag{6}$$

The observed outcomes are taken as a discretization of $\mathbf{X}_g^*$ through a set of threshold parameters ν_g^*. For an ordinal component X_g of $\mathbf{X}_g$ with c categories (levels), the relationship between the observed scores and the latent response variables is

$$X_g = m \quad \text{if} \quad \nu_{gm} \leq X_g^* < \nu_{g(m+1)}, \tag{7}$$

where $\{\nu_{g0}, \nu_{g1}, \ldots, \nu_{g(c+1)}\}$ are the threshold parameters and $\nu_{g0} = -\infty$ and $\nu_{g(c+1)} = +\infty$ by definition. In this model, both the latent factors and the latent response variables are not observed and so their origins and scales are not known. Therefore, in addition to the constraints necessary for identifying the distributions of the latent factors, it is also necessary to specify additional constraints for fixing the distributions of the latent response variables, because it is not possible to determine the thresholds and $(\mu_g^*, \mathbf{\Sigma}_g^*)$ simultaneously. If the origin and scale of the components of $\mathbf{X}_g^*$ are shifted and rescaled, and their respective thresholds transformed likewise, the new latent response variables and thresholds will yield the same probability structure of the observed scores of $\mathbf{X}_g$. Therefore, it is necessary to specify constraints on the thresholds, the parameters of the latent response variables, or both, in order to identify the latent response distributions [29,84]. This in turn changes the invariance constraints that must be imposed for testing measurement invariance.

In the present work, we followed the approach described in Wu and Estabrook for setting the identification conditions and invariance constraints [84], as described below.

In the configural models we identified the location and scale of the latent response variables and the common factors by setting their means and variances to 0 and 1,

respectively, for all groups. This is the default method used in `lavaan`. The conditions described in Millsap and Yun-Tein [29] are different because the locations and scales of the latent response variables are fixed by imposing conditions on the thresholds. The two methods are equivalent for the configural model.

Wu and Estabrook described several possible sequences for testing the successive invariance levels ([84], Fig 1). The simplest alternative is to start by fixing the thresholds across groups. This fixes the locations and scales of the latent response variables, after which the model with ordinal items is reduced to one involving the (already identified) continuous latent responses. The sequence (and hierarchy) of the invariance constraints then follows as for the continuous case. More specifically, fixing the thresholds reduces eq.(4) to eq.(3) of [84]. This note clarifies our specification of the invariance constraints at different levels of invariance, shown in S9 Table.

For testing metric invariance, we required the thresholds and loadings to be invariant across groups. This ensured invariance of the scales of both the latent response variables and the common factors. For scalar invariance, we required invariance of the thresholds, loadings and intercepts, thus fixing the locations and scales of both the latent response variables and the common factors. Wu and Estabrook also showed that when threshold and loading invariance is imposed, the intercepts of the latent response variables need to be freed for all groups except the reference group, because otherwise the resulting model would be over-identified [84]. In this work, we used these identification conditions, as implemented in the `semTools::measEq.syntax()` function [58].

When metric invariance holds, the variance-covariance matrix of the latent variables can be tested for invariance across the groups; when scalar invariance also holds, the latent means can also be tested for invariance [15]. S9 Table summarizes the invariance constraints for each level of measurement and structural invariance.
